# Electoral rewards for political grandstanding

**DOI:** 10.1073/pnas.2214697120

**Published:** 2023-04-18

**Authors:** Ju Yeon Park

**Affiliations:** ^a^Department of Government, University of Essex, Colchester CO4 3SQ, United Kingdom

**Keywords:** congress, committee hearing, political speech, grandstanding, election

## Abstract

Many assumed that legislators send political messages or even grandstand in expectation of gaining electoral rewards. However, largely due to a lack of proper data and measurements, this assumption has not been tested. Publicized committee hearings provide a unique environment to consistently observe changes in legislators’ speech patterns and test this assumption. Using House committee hearing transcripts from 1997 to 2016 and Grandstanding Scores–which capture the intensity of political messages conveyed in members’ statements in hearings–I find that an increase in a member’s messaging efforts in a given Congress leads to increased vote share in the following election. This suggests that legislators’ grandstanding remarks, often regarded as cheap talk, can be an effective electoral strategy. Additional findings suggest that PAC donors respond differently to members’ grandstanding behavior. Specifically, while voters react to members’ grandstanding positively but are ignorant about their legislative effectiveness, PAC donors are unmoved by members’ grandstanding behaviors and reward members’ effective law-making activities instead. These asymmetric reactions from voters and donors may provide members with a twisted incentive to appeal to voters merely by making impressive, political speeches while legislating in favor of organized interests, which raises concerns about how representative democracy works.

## Significance

In many representative democracies, legislators sometimes focus on making political points rather than making policy. Previous studies assumed or argued that they do so in an expectation of gaining more votes in the following election, but this claim has not been studied systematically. By looking at data on legislators’ statements, this paper demonstrates that US House representatives who made statements conveying political messages more intensely in any given two-year term tended to gain higher vote shares in the following election. In contrast, organized donors did not react to legislators’ political speeches but rewarded their legislative achievements instead. This raises concerns as legislators may represent organized interests when making policy while pleasing voters only by giving impressive, political speeches.

On October 5, 2021, in a US House oversight hearing on government responses to hurricane Ida, Representative Ralph Norman, a Republican from South Carolina, said the following when given a chance to question Deanne Criswell, an administrator of the Federal Emergency Management Agency: “Ms. Criswell, you talk about ‘crisis’ ‘we’ve got a crisis on the border... We’ve got an inflation crisis... We’ve got a military crisis in Afghanistan... We’ve got crises, and this administration has simply not addressed them.” He chose to give an acerbic partisan speech, which was completely off the topic, using his limited time to talk rather than to gain information from the administrator. Congressional observers report that members’ grandstanding, as in this example, is an everyday phenomenon (1). Why do members invest time in writing and delivering such political speeches?

In many representative democracies, lawmakers often make political, symbolic statements when given the opportunity to deliver a public speech. As legislators are generally believed to be single-minded reelection seekers (2), scholars have argued that they do so with an expectation of improving their reelection prospects (3, 4). Yet, we do not have a clear understanding of whether and how legislators’ messaging activities increase their reelection prospects. This may be primarily due to the lack of high-quality data that allow measuring legislators’ messaging efforts as they change over time. In the United States, for example, intensified partisan competition induced members of Congress to increasingly focus on sending political messages rather than making policies (5). Increasing polarization in Congress cultivates greater incentives for members to send political messages, also known as “grandstanding” (6). Thus, assessing the electoral effects of members’ messaging efforts is essential and timely, and this study undertakes the analysis by examining whether US House representatives’ messaging efforts are electorally rewarded by their increased vote shares.

This paper presents one primary argument: The political messages that legislators communicate publicly are likely to have electoral consequences. For politicians to stay in office and be reelected, winning the hearts and minds of voters is key. Voters want politicians to represent their policy preferences (7), but they often lack the ability to access or assess information about their representatives’ legislative activities (8). Consequently, voters tend to rely on their representatives’ explanations of their own legislative achievements, which they are likely to selectively communicate to satisfy constituents’ interests, or media reports about their performance. In general, the media tend to disproportionately highlight members’ symbolic, grandstanding statements, thereby conveying political messages that are easier for voters to understand and adopt (9).

Political messages typically conveyed in grandstanding statements tend to contain elements resembling electoral campaign messages such as position taking, advertising, and credit claiming. For example, in committee hearings, these statements are likely to involve taking positions on policy issues on subjective grounds, criticizing the other party or its members, praising the achievements of one’s own party, or attacking a witness while asking questions. I define this set of speech styles as “grandstanding.” Hence, once voters are successfully exposed to these messages, the effects of members’ grandstanding on voter behavior will be similar to those of campaign messages aimed at strengthening constituents’ support. Therefore, members’ grandstanding efforts are likely to result in an increased vote share in the following election. In addition, the effect size will depend on the level of exposure, which can be increased by the salience of a hearing or decreased by redistricting that disconnects the relationship between the incumbent and their constituents. Therefore, I propose to test the following two hypotheses:

**Vote Share Hypothesis:** As a member grandstands more in any given congress, they are likely to gain a higher vote share in the following election.
**Exposure Hypothesis:** A member’s grandstanding tends to have a greater effect on their vote share when their constituents are better exposed to their grandstanding.

My analysis focuses on changes in members’ speech patterns during congressional committee hearings. Committee hearings provide a unique environment that facilitates the analysis of legislators’ strategic allocation of their behavioral focus. Committee members can achieve multiple competing goals in public hearings (10). For example, they can collect expert policy-relevant information (11); at the same time, hearings provide members with free airtime to promote their policy perspectives, communicate their voting intentions, and gain media attention (12).^*^ Given that members have limits on the amount of time they may speak in hearings, they must choose how to strategically allocate this time (e.g., whether to focus on policy details or to send electoral messages) (6). These institutional features of committee hearings are absent from other publicized communication channels. Thus, changes in members’ speaking styles demonstrated in these hearings provide ideal data to learn about any modifications in their priorities. Moreover, members are likely to apply these changes simultaneously to activities they engage in outside the context of committee hearings.

To test my theory, I use an original dataset on House committee hearing transcripts from the 105th to 114th Congresses and the “Grandstanding Score,” which measures the intensity of the political messages conveyed in each statement made by committee members (13). Previous research on legislators’ behavioral styles introduced various ways to classify them into multiple categories (14, 15). However, few studies quantified legislators’ messaging or posturing efforts as a continuous variable. The Grandstanding Score is the first instance of such a measurement.^†^ Using hearing transcript data, I construct a member-level panel dataset and link it to the data on election results.

Major findings from this analysis suggest that when a member engages in more messaging activities in any given congress than they did in other congresses they served, their vote share tends to increase. However, members’ effectiveness in legislative activities (17) does not have any effect on their vote share. This finding–that politicians’ grandstanding efforts have a substantial, positive effect on their vote share–is striking. Also, it resonates with the classic discussion on work horses versus show horses, where Payne (15) regrettably states, “the most capable baseball players are the most publicized,” while the same relationship does not seem to hold for legislators.

As a complementary analysis, I present a test of whether organized groups react to members’ grandstanding in hearings. Studies have found that organized interest groups tend to react to what members say in hearings. For example, members’ engagement in analytical discourse—as opposed to symbolic, experiential messages—is rewarded through hard money contributions from political donors (18). Furthermore, members speaking similarly with witnesses who represent interest groups are likely to be offered jobs at lobbying firms once they leave Congress (19). Consistent with previous results, I find that organized donors are unlikely to be moved by members’ political cheap talk but reward members’ effective lawmaking activities instead, which contrasts with voters’ reactions.

This paper provides several interesting implications. First, making political speeches intensely and repeatedly can be an effective electoral strategy in any representative democracy where lawmakers are able to make public speeches and run for reelection. Second, the asymmetric reactions of voters and donors toward members’ grandstanding behavior may incentivize members to appeal to voters by making impressive political speeches while legislating in favor of organized interests, which may pose a threat to the sound functioning of representative democracy. Third, the findings of this study suggest that members may want to exploit committee hearings to make points rather than making policy. In this regard, legislators’ electoral incentives may work at the cost of institutional effectiveness, which is consistent with previous studies (20).

## Data and Methods

I use my original dataset on House committee hearings transcripts (13) as it is especially well suited to test my hypotheses. The data are available in two versions: at the statement level and at the member level. The statement-level dataset includes 1,026,677 statements committee members made in 12,821 House committee hearings from the 105th to the 114th Congresses as well as variables that characterize the congresses, committees, hearings, and individual members. Most importantly, the dataset includes “Grandstanding Scores,” which captures the intensity of political messages conveyed in each statement or utterance that committee members made in these hearings.

The Grandstanding Score was constructed through a crowd-sourced supervised learning method. Measuring a latent trait using a supervised learning method typically requires human coding. Studies report that crowd-sourced human coders tend to generate more consistent and reproducible codes than several trained coders (21, 22). Therefore, I used Amazon Mechanical Turk (MTurk) workers to provide coding for a subset of the data using the SentimentIt R package (It is renamed labelR) (22). The package helps create text-coding tasks on the MTurk platform through R and analyze them. In particular, it facilitates measurement of the relative intensity of a latent trait in text data through repeated pair-wise comparisons of randomly paired texts. The product it generates is a continuous measurement of the latent trait for the compared texts. This measurement scheme is distinct from other conventional methods of manually classifying each text into one of the preset categories.

Coding was conducted through the following procedure. First, 3,000 paragraphs from member statements were randomly chosen to construct the sample data to be human-coded.

Second, the coders were provided detailed instructions on coding schemes and the task itself. A statement is defined as grandstanding if it denounces or praises a person or an institution, takes a policy position on subjective grounds, or asks questions to embarrass or attack a witness (To further clarify the definition of grandstanding statements, explanations of what is not grandstanding were also provided. A typical nongrandstanding statement offers an objective description of a policy-relevant situation or questions a witness to uncover facts or seek an expert opinion). The instructions also included multiple examples of grandstanding and nongrandstanding statements. The task was to choose the paragraph that sounded more like grandstanding between two randomly paired paragraphs.

Third, for training and screening purposes, the workers were asked to answer five practice questions and six test questions on the Qualtrics platform. Upon submitting their answers to each question, they were provided the correct answer and a supporting explanation for the answer. Only the workers who answered five out of six test questions correctly were hired; that was 169 out of 387 workers.

Fourth, the entire set of tasks involved 30,000 pairwise comparisons where each of the 3,000 sample paragraphs appeared 20 times, and workers earned $0.08 for answering each comparison.

Fifth, based on the binary choices that workers made in all 30,000 comparison tasks, each sample statement was assigned a score using a Bradley–Terry model. This is a Bayesian model that is embedded in the SentimentIt package and estimates the probability that a document *j* will be chosen when compared with another document *i* by a worker *k* using Hamiltonian Markov chain Monte Carlo sampling. See the model specification and the priors for key parameters below.

$$\begin{array}{c} \begin{array}{c} Pr(y_{ijk}=j)=\frac{exp(b_{k}(a_{j}-a_{i}))}{1+exp(b_{k}(a_{j}-a_{i}))}\\ a_{j}∼N(0,1)\;b_{k}∼trN(0,\sigma^{2})\;\sigma ∼trN(0,3), \end{array} \end{array}$$

where *N* denotes the normal distribution, and *trN* denotes the normal distribution truncated at zero to allow positive values only. The model produces the latent trait of each document, *a*_*j*_, and each worker’s quality, *b*_*k*_ (For more information about the model, Carlson and Montgomery (22) and Carpenter et al. (23)). The resulting score for the 30,000 sample texts runs from −2.4 to 2.6.

Using the sample paragraphs as a training set, machine learning models were fit to generate the Grandstanding Score for all member statements in the data. The process for machine learning and prediction is as follows: First, the entire corpus was put through a set of preprocessing steps: tokenizing, stemming, lower-casing, and removing punctuation and stop words.

Second, to be fed into machine learning models, the text data were transformed into document-level matrices using three approaches: plain bag-of-words, term frequency-inverse document frequency (tf-idf), and doc2vec.

Third, the 3,000 human-coded sample paragraphs were randomly divided into two sets: 2,700 for training models and 300 for validating the final model.

Fourth, on the 2,700 sample paragraphs, 13 machine learning model specifications were fit on each of the three document-level matrices generating a total of 39 machine learning model predictions for each member statement in the corpus.^‡^ Basically, in each model, the human-coded scores served as a dependent variable, and the information in a document-level matrix for these paragraphs served as predictors.

Fifth, an ensemble Bayesian model averaging technique (24) was used to aggregate the 39 models and optimize the prediction performance of the final model. Only nine models received nonzero weights and together formed the final model. When the final model predicted the scores for the 300 paragraphs set aside from fitting machine learning models, the correlation between the predicted scores and human-coded scores was 0.703, and the root mean squared error between the two was 0.613. *SI Appendix*, section 2 the list of nine selected models, further statistical and substantive validation of the final model, and its resulting score including the five most and least grandstanding statements.

**Fig. 1.**
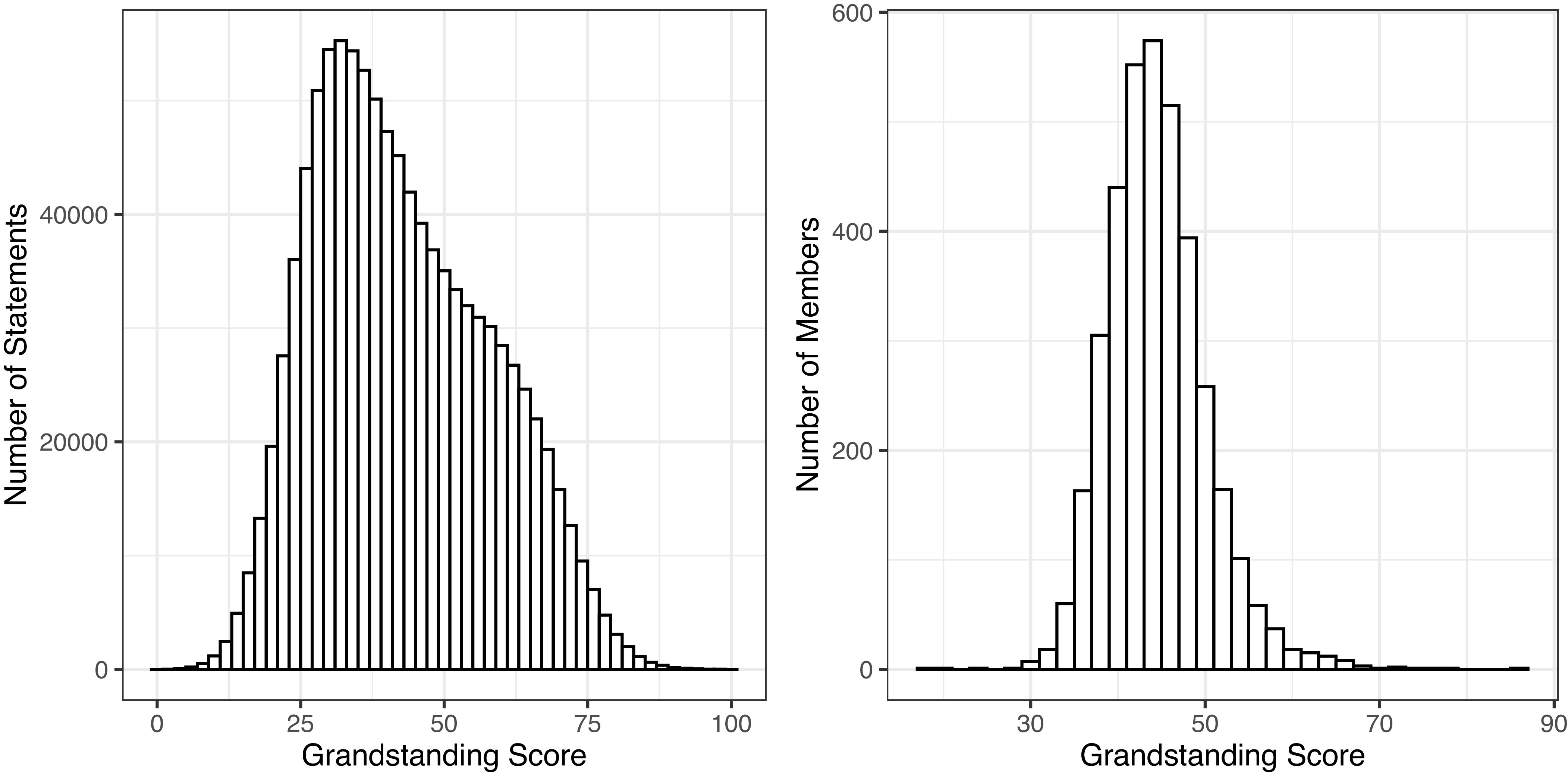
Distribution of the Grandstanding Score.

Lastly, the final model was used to predict the score for each member statement in the data, and the measurement was rescaled to run from 0 to 100. The mean of the Grandstanding Score is 53.48, and its SD is 15.22. Then, the statement-level data were aggregated by member and congress to construct member-level data that are panel data. In the member-level data, the score is averaged for each member in each congress and ranges from 17.65 to 85.78 with its pooled SD at 4.34 (The pooled SD is the weighted average of standard deviations of each member’s scores across the congresses in which they served). Fig. 1 presents the distribution of the score in each dataset. Park (13) for more information on the Grandstanding Score.

To test the vote share hypothesis, I use the member-level panel data in which the Grandstanding Score is the average score of each member in each congress. As a dependent variable, I compute the percentage of votes that a member received in the election held at the end of a given congress in which the member’s Grandstanding Score is measured (For example, the effect of the Grandstanding Score measured from a member’s statements during the 110th Congress (2007-2008) on their vote share in the 2008 election is analyzed). The election result data are collected from the CQ Press.^§^

In the regression analysis, I control for several sets of potential confounders addressing members’ electoral conditions, institutional status, and behavioral patterns demonstrated in a given congress. First, I control for the total number of statements a member made in hearings in each congress with binary indicators for party leaders and committee leaders. This is because as a member makes more statements in public hearings or serves in a leadership position, their publicity and thus their vote share may increase. As voters may vote based on members’ legislative achievements from a given congress, I control for their Legislative Effectiveness Score (LES).^¶^ The level of loyalty to the party, captured by the party unity score,^#^ may increase support from the party leadership for the member through endorsement or allocation of campaign contributions and thus boost their vote share. Because they can positively affect the vote share, I also control for members’ seniority and its squared term, the electoral security of the incumbent measured by taking the absolute value of (the Democratic presidential candidate’s two-party vote share in the previous election - 0.5), and the ideological intensity which is the absolute value of the first dimension of the DW-NOMINATE score. Indicators for being a minority party member and for unified government, and the interaction between the two, are included because of their importance in determining members’ incentives to grandstand and in shaping the electoral environment in general (Park (13) finds that minority party members tend to grandstand more in general and more so under a unified government). The challenger quality capturing whether the challenger has held previous elected office (25) and the ratio of campaign spending between an incumbent and their challenger are controlled as they are important electoral conditions shaping the election results. To address the midterm effect, I include an interaction between the indicators for midterm elections and for the president’s party members. Another important factor that may disconnect the electoral connection between a member and constituents is redistricting. Thus, an indicator for redistricting is included.^||^

In addition, to make their effect size comparable to each other, some variables are rescaled to range from 0 to 1 if they do not originally. The rescaled variables are the ideological intensity, LES, seniority, and statement frequency.

For the analysis, I estimate within-individual effects by fitting an OLS regression with member fixed effects and congress fixed effects with robust standard errors clustered by member.^**^ So, the effects of explanatory variables should be interpreted in terms of how a unit change in these variables affects the same member’s change in vote share while holding individual-specific, time-invariant unobserved variables constant. Congress fixed effects control for time-varying, congress-specific features. Although estimating within-individual effects by using panel data with unit-fixed effects helps control for unit-specific time-invariant unobservables, there is a limitation to making causal claims from this analysis plan. Thus, findings presented in this study are correlational rather than causal.

To test the exposure hypothesis, I use the statement-level data because, unlike the member-level data, they allow for testing important characteristics of hearings such as salience of a hearing which is key to the hypothesis. I measure the level of voters’ potential exposure to members’ grandstanding statements based on two indicators: the salience of a hearing and redistricting. First, the effect of members’ grandstanding can be amplified when it occurs in a salient hearing because the media is more likely to cover hearings on salient issues, and thus, the public will be better exposed to these hearings. The level of issue salience of a hearing is indirectly measured by counting the number of committee members who attended and spoke in a hearing and rescaled to run from 0 to 1. This variable can capture the salience of a hearing because members would have a greater incentive to speak in a hearing in which they expect more media attention.^††^ To address this moderating effect of issue salience, I add an interaction term between the Grandstanding Score and the level of salience with the full set of control variables. Second, the indicator for redistricting is coded as one if a member experienced redistricting in a given Congress; 0 otherwise. Similar to the test on the moderating effect of the salience of a hearing, in a separate regression, I interact the Grandstanding Score with the indicator for redistricting to test the moderating effect of redistricting.

In the statement-level analysis, I use an OLS model with fixed effects for committees and congresses and random effects for members and hearings.^‡‡^ As the statement-level data only allow cross-sectional comparisons, to control for variations in the vote share across members, I include members’ vote share from the previous election and its squared term as its serial relationship may not be linear.

## Results

### 1. Main analysis: The Effect of Grandstanding on Vote Share.

The first two models in Table 1 present regression results testing the effects of members’ grandstanding on their vote share in percentages. The first model is a baseline model that includes the Grandstanding Score as the only covariate while the member fixed effects and congress fixed effects are included. The second model controls for other potential confounders. Most of the control variables are omitted from this table for brevity of presentation, but the full results are available in *SI Appendix*, Table S3. In both baseline and full model specifications, the Grandstanding Score has a positive and statistically significant coefficient providing support for the vote share hypothesis. That is, if a member grandstands more in a given congress than they did in other congresses, they are likely to gain a higher vote share in the following election. More specifically, based on the second model with full specification, a one-point increase in the Grandstanding Score is likely to increase the vote share by 0.07%. Compared to previous findings on campaign effects (26, 27), it is a surprisingly large effect;^§§^ for example, given the findings by Spenkuch and Toniatti (27), it is equivalent to the effect of broadcasting almost three more TV advertisements in favor of the incumbent.

**Table 1.** The effect of Grandstanding on vote share (%) and PAC contributions ($1K)

	*Vote share (%)*		*Vote share (%, Lag)*	*PAC contributions ($1K)*	
	Model 1	Model 2	Model 3	Model 4	Model 5
Grandstanding score	0.098^**^ (0.043)	0.070^**^ (0.031)	−0.001 (0.042)	0.367 (1.166)	0.692 (1.392)
Legislative effectiveness		−2.434 (1.690)	−0.754 (2.396)		591.541^***^ (122.577)
Constant	56.884^***^ (2.188)	36.031^***^ (5.328)	49.692^***^ (6.218)	382.819^***^ (44.140)	424.763^**^ (197.052)
Other controls	-	✓	✓	-	✓
Member effect	Fixed	Fixed	Fixed	Fixed	Fixed
Congress effect	Fixed	Fixed	Fixed	Fixed	Fixed
Observations	3,259	2,401	2,471	3,183	2,474
Adjusted R^2^	0.504	0.748	0.651	0.607	0.631

Note:^*^P< 0.1; ^**^P< 0.05; ^***^P< 0.01

In the first two models, the dependent variable is members’ vote share in the election held at the end of their two-yearterm. In the third model, it is the vote share from the previous election. In the last two models, it is the total receipt of PAC contributions each member received during their two-year term. Other control variables are omitted but are available in *SI Appendix*, Table S3. Robust standard errors clustered by member are in parentheses.

Is there any chance that members tend to grandstand more if their constituents become more supportive of them or their party so that they expect to have easier elections? If so, the direction of the effect may run in the opposite direction. To address this concern, Model 2 controls for changes in the strength of the partisan leaning of districts based on the most recent presidential elections, and I showed that the Grandstanding Score still affects members’ vote share positively. In addition, as a placebo test, Model 3 replaces the dependent variable with members’ vote shares from their previous elections to check whether a member’s grandstanding in their current term (e.g., 110th Congress) is related to their vote share in the election held at the end of their previous term (e.g., 109th Congress).^¶¶^ If there is reverse causality, the coefficient on the Grandstanding Score has to be positive and significant in this model as well, but it is not. This suggests that the vote share hypothesis is correct about the direction of the effect.^##^

**Fig. 2.**
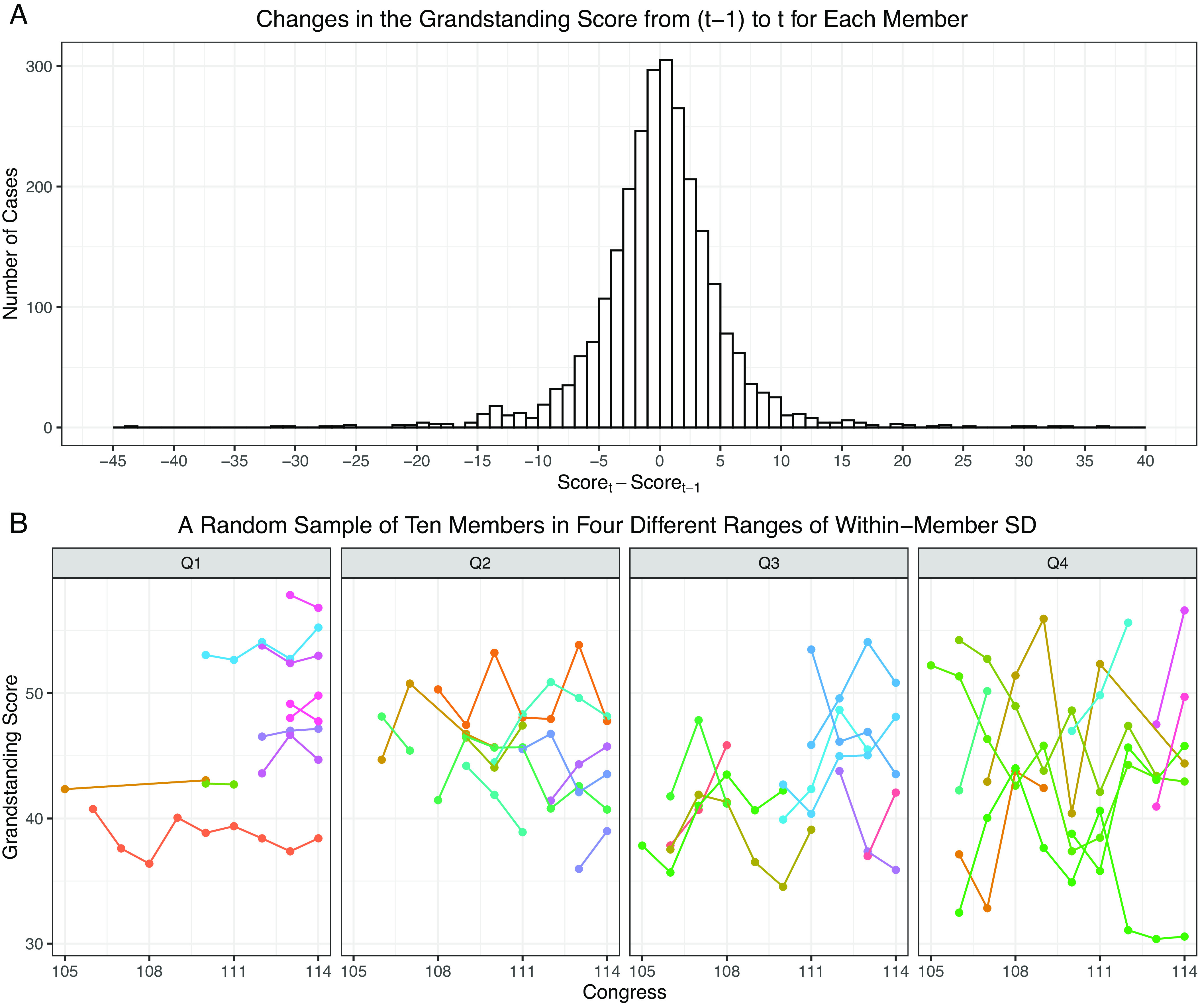
The distribution of changes in the Grandstanding Score. (A) Changes in the Grandstanding Score from (t-1) to t for Each Member. (B) A Random Sample of Ten Members in Four Different Ranges of Within-Member SD.

Now, more realistically, how much do members change their speech styles from one congress to another in general? To answer this question, I compute the difference in the average Grandstanding Score between one term and its preceding term for each member. The distribution of this difference is shown in Fig. 2*A*. In most cases (72.22%), members change their Grandstanding Score by one pooled SD of the score in the member-level data, which is 4.34, or less. There is, however, a significant proportion of members (27.78%) who changed their speech styles by more than one pooled SD ranging up to over 20 points in extreme cases.

Fig. 2*B* further illustrates over time changes in 40 members chosen through a random block sampling. The sampling procedure is as follows: I computed a SD using the overtime variations in the Grandstanding Score of each member who appeared more than once in the data. Then, I randomly sampled ten members from each quartile of the distribution of these member-specific standard deviations. In the graph, the Grandstanding Scores of the same member are connected in a line. The graph presents ten lines in each of the four panels. We can see that each member’s Grandstanding Score varies over time without any consistent pattern, and obviously, the variation is larger in the last panel, which shows members with standard deviations falling in the top quartile. These two graphs in Fig. 2 together demonstrate that there is a good amount of overtime changes in individual member’s speaking patterns to be explored in the data.

Members’ incentives to grandstand may vary depending on various institutional conditions. Certain members may experience a greater need to change their speaking styles (For more information on this, Park (13) which specifically tests under what conditions members tend to grandstand more in committee hearings) and those members who changed their behavior drastically are of interest in this study. Then, the result suggests that if a member grandstands more by 10 points, their vote share is likely to increase by 0.7%, and a 20-point increase is likely to lead to an increase in vote share of 1.4%. Whether this amount of increase would significantly improve a member’s reelection prospects depends on their electoral conditions, but it is certainly a substantial effect.

For example, when we look at only the members who made at least 10 statements in a given congress and whose Grandstanding Score jumped by more than 10 points from the previous congress, they experienced a 6.6% increase in their vote share on average. One of them is former Vice President Mike Pence who served as a House representative from Indiana from the 107th to 112th Congresses. In his second term, his Grandstanding Score increased from his first term by 11.89%, and he gained 3% more votes at the end of his second term than he received at the end of his first term. Similarly, Mac Thornberry, who served as a representative of Texas’ 13th district from the 105th to 114th Congresses, experienced an 11% increase in his vote share at the end of the 107th Congress after his Grandstanding Score increased by 13.73% compared to that in the 106th Congress.

**Fig. 3.**
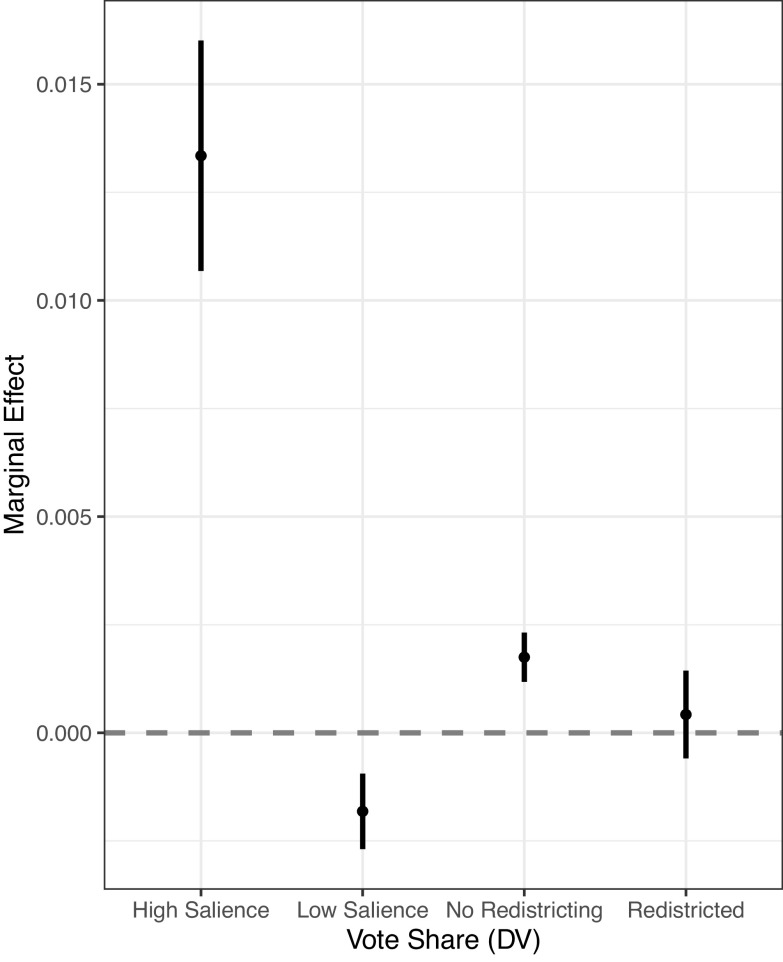
Marginal effects of the Grandstanding Score by salience or redistricting.

Next, I test the exposure hypothesis using two measures of the potential exposure of members’ grandstanding statements. The regression results are presented in *SI Appendix*, Table S4 in section 3.A. The marginal effects of the Grandstanding Score when the level of salience is highest (1) and lowest (0) are presented as the first two dots in Fig. 3. In the most salient hearing where the largest number of committee members attended and spoke, making a 100-point grandstanding statement is likely to increase the percentage of votes by 1.3%. But this positive effect turns negative in less salient hearings, probably because it induced a backfire effect from nonvoter audiences attentive to this type of hearings. This result confirms the expected moderating effect: A member’s grandstanding is likely to have a greater effect on their vote share when performed in a more salient hearing.

Second, I also test whether the effect of grandstanding is reduced when the incumbent experienced redistricting during their term. Redistricting disconnects the link between some voters and the incumbent. In particular, constituents newly included in an incumbent’s district might not have been sufficiently exposed to the incumbent’s political messages before the election. Thus, the effect of grandstanding may reduce when redistricting occurs due to the lack of exposure. I test the moderating effect of redistricting by interacting the Grandstanding Score with the indicator for redistricting. The coefficient on the interaction term is negative and statistically significant, −0.001 (s.e. = 0.0006), and the coefficient on the Grandstanding Score is positive and significant, 0.002 (s.e. = 0.0003). The marginal effects of the Grandstanding Score in redistricted and nonredistricted cases, respectively, are presented as the last two dots in Fig. 3. The result suggests that in general, the effect of members’ grandstanding exists only when there is no redistricting. In summary, these additional tests provide support for the exposure hypothesis.

Through what mechanism would members’ grandstanding increase their vote share? Would it be driven by better mobilizing incumbents’ copartisans or by persuading independents and out-partisans to switch their votes in support of the incumbent? I conduct a further analysis using three-wave panel survey data from the Cooperative Election Study (CES) from 2010 to 2014 and find that a member’s messaging effort tends to increase their vote share due to increased support from previous nonsupporters rather than due to increased turnout among their base supporters. *SI Appendix*, section 4 presents the analysis. However, since this is not a randomized experiment, its findings are only correlational and future research with enhanced data and research designs may confirm the causal path of these microlevel mechanisms.

### 2. Additional Analysis: The Effect of Members’ Grandstanding on PAC Contributions.

Political donors are another type of audience of congressional committee hearings. How would they react to members’ grandstanding behavior? Previous studies find that interest groups tend to reward committee members’ engagement in analytical—as opposed to experiential or symbolic—discourse in hearings through making political contributions (18). Given that grandstanding statements tend to be symbolic rather than analytical about policy details, I expect that organized interests would not react to members’ grandstanding efforts.

In this analysis, the dependent variable is the total receipt of PAC contributions for each member in each election cycle in thousand dollars. In addition to the set of controls included in the analysis on the vote share, the number of candidates running for the district primary is controlled because it may reduce the amount of donations an incumbent can receive due to intensified competition. Both variables are from Bonica’s campaign finance data for congressional elections (28).

Models 4 and 5 in Table 1 present key results from the regressions testing this expectation using member-level data. The coefficients on the Grandstanding Score are insignificant in both the baseline and full models. This result suggests that members’ grandstanding does not affect the PAC contributions that they receive.

Another interesting finding is that the LES has a positive and statistically significant effect on political donations while it had no effect on the vote share as shown in Models 1 and 2. This finding highlights a stark contrast between voters and PAC donors regarding the types of members’ activities to which they pay attention. That is, voters are more likely to react to members’ political statements while being relatively ignorant about their legislative achievements. In contrast, organized interests barely react to members’ political statements; instead, they attend to members’ legislative activities and assess those when making their donation decisions.

This asymmetric reward scheme raises a concern about the representation of constituents’ preferences because it incentivizes politicians to win voters’ minds only by making impressive political speeches while proposing and voting on bills that reflect organized interests’ preferences instead of public welfare if those goals do not coincide.

## Discussion and Conclusion

This study investigates whether legislators’ messaging activities are electorally rewarded. Using House committee hearing transcript data and panel data analysis, the study finds that members’ messaging efforts tend to increase their vote share in the following election. It is surprising that legislators’ grandstanding remarks—which most people consider theatrical, political cheap talk—do have electoral consequences. This finding suggests that not all grandstanding statements are cheap talk, at least from voters’ perspectives.

Contrary to voters, however, PAC donors do not react to members’ grandstanding statements and instead reward their effective lawmaking activities, about which most voters are ignorant. These asymmetric reactions from voters and PAC donors to members’ grandstanding behavior and legislative effectiveness raise concerns about representation. Since politicians can win voters’ minds only by making impressive political statements while enacting policy benefiting special interests, the preferences of special interests are likely better represented in public policy than those of the general public. This conjecture is consistent with the literature that shows politicians’ voting patterns to be more aligned with their donors’ preferences than with that of their constituents (29). Therefore, unless voters are better trained to acquire information about members’ legislative activities and achievements and develop an ability to assess that information, this asymmetric representation of voters and donors may persist. It is important to note that this finding and implication can be broadly applied to any representative democracies where legislators have a similar incentive structure.

This paper paves the way for multiple avenues of future research. First, although the current analysis used panel data with member fixed effects to control for member-specific time-invariant confounders, this research design cannot completely remove the potential that individual-level time-varying unobservables may confound the key relationship found in this study rendering these findings essentially correlational. Thus, future research with an enhanced research design (e.g., a randomized experiment) could be worthwhile to examine whether these findings are causal. Another line of research is to examine how donation patterns of different types of political donors (e.g., individual donors versus PAC donors; district donors versus out-of-district donors) are affected by members’ messaging activities. In addition, while this analysis focused on electoral rewards from voters, investigating nonelectoral rewards, such as a position in a higher office or any other career benefits that members gain from grandstanding, would be another interesting extension of this study.

## Supplementary Material

Appendix 01 (PDF)

## Data Availability

Rdata have been deposited in Harvard Dataverse (https://doi.org/10.7910/DVN/PM4YOL) (30).

## Footnotes

^*^*SI Appendix*, section 1 provides an analysis showing how member statements in hearings can reach the public using the data on the various public communication channels that lawmakers use, including newspaper articles, social media, etc.

^†^Using members’ press releases, Grimmer (16) introduced a continuous measurement of members’ messaging styles: whether one tends to claim credit as opposed to advertising or position taking. Their measurement differs from the one used in this study: The latter measures members’ tendency to focus on political messaging as opposed to other productive, legislative activities; the former captures various messaging styles once a member engages in messaging activities.

^‡^The models include Random Forest, Lasso, Support Vector Machine, Gradient Boosting Machine, Discrete Bayesian Additive Regression Tree sample, Bayesian Generalized Linear Model, and Linear regression. For some models, more than one specification with different tuning parameters was estimated.

^§^https://library.cqpress.com/elections/.

^¶^The LES captures how many bills a member introduced in a given congress and how many of them received action in committee, received action on the House floor, passed the House, and were enacted into law. In addition, its measurement process assigns more weight to more important bills. Its correlation with the Grandstanding Score is −0.125. Volden and Wiseman’s work (17) for more detailed measurement procedures.

^#^The party unity score measures the frequency of voting with the party median on party unity votes and is collected from VoteView.com.

^||^The data on redistricting for each district were collected from the CQ Press.

^**^In the model, I do not include a lagged dependent variable because Durbin–Watson tests of autocorrelation on the models presented in Table 1 provide insignificant test statistics suggesting no autocorrelation in the dependent variable. Even if a lagged dependent variable is included, the coefficient on the Grandstanding Score barely changes: 0.067 (s.e. = 0.031).

^††^Alternatively, the salience of a hearing can be measured by the frequency of the media coverage on a hearing, but this approach is problematic because it can be a result of members’ grandstanding behavior rendering it a posttreatment variable. In contrast, the number of members speaking in a hearing is less likely to suffer from this problem and thus is a better measurement.

^‡‡^The main reason for not including fixed effects for members and hearings is due to the large number of fixed effect coefficients to be estimated, which prevents R software from successfully fitting the model.

^§§^Spenkuch and Toniatti (27) find some campaign effects in their natural experiment on a US presidential election utilizing television advertisement viewership that varied exogenously across neighboring counties. They find that if potential voters in a county are exposed to 21 more TV advertisements in favor of a Democratic candidate than advertisements in favor of a Republican candidate, the Democratic candidate’s vote share is likely to increase by 0.5%.

^¶¶^The coefficients on most of the control variables including this variable are omitted from Table 1 but are presented in *SI Appendix*, Table S3. Ideally, it is better to have the Grandstanding Score as a dependent variable and see whether members’ previous vote share predicts the score. Park (13) presents this result using the same member-level panel hearing data with member fixed effects and congress fixed effects with robust standard errors clustered by member, just as in this study. She finds that the previous vote share and its squared term do not influence members’ Grandstanding Scores. Model 2 in Table 2 in her paper, p.223.

^##^Furthermore, in case there are any other member-specific time-varying unobservable confounders that are not captured by the list of control variables, I also control for unit-specific linear time trends and present results in *SI Appendix*, section 3.B and find mixed results. However, the results presented in Table 1 provide clear evidence for the correlational effect of members’ messaging efforts on their vote share in the following election, but not the other way around.
