# Supplementary material for: Electoral rewards for political grandstanding
Source: Proc Natl Acad Sci U S A. 2023 Apr 18;120(17):e2214697120. doi: 10.1073/pnas.2214697120 (PMC10151507; doi:10.1073/pnas.2214697120)
Supplement: Supplementary file 1 — Appendix 01 (PDF) [file pnas.2214697120.sapp.pdf]

Supporting Information for

Electoral Rewards for Political Grandstanding

Ju Yeon Park

E-mail: jp20761@essex.ac.uk

This PDF file includes:

Supporting text
Figs. S1 to S3
Tables S1 to S9
SI References

Supporting Information Text

1. Committee Hearings as a Constituent Communication Channel

One may think that ordinary voters barely watch hearings on C-SPAN. There are, however, a variety of communication channels through which members can make what they said in hearings better reach the public.

First, I analyze newspaper articles as they are one of the major news outlets that members' official activities are exposed to the public. For the analysis, I utilize news articles published by the Washington Post in 2019 and randomly selected ten days.* Figure S1 presents the news articles on hearings and members' grandstanding statements reported in the Washington Post on the randomly selected ten days by date. Using keyword search by "committee hearing", I found 97 articles that cover stories about congressional hearings. 35 of them quote or cite members' statements from a hearing, and in all these articles at least one statement is characterized as grandstanding.

Table S1 lists members' example grandstanding statements quoted in newspaper articles at the Washington Post on the ten randomly selected days –a day per month– in 2019 while Congress was in session. I picked one statement a day from many grandstanding statements quoted on each day. This suggests that what members say in committee hearings is frequently publicized for broad public consumption.

Table S1. Example Grandstanding Statements Quoted in the Washington Post News Articles

Date	Member	Grandstanding Statements
Feb. 18	-	-
Mar. 13	Sen. Josh Hawley (R-MO), Armed Services	Josh Hawley (R-Mo.) is still the new guy on Capitol Hill. But the freshman senator is swiftly emerging as one of the Republican Party's toughest critics of Big Tech. Hawley's rigorous grilling of Google executive Will DeVries was the most heated exchange during yesterday's privacy hearing: He slammed Google for collecting people's location data on Android phones — even after they try to disable the tracking function. He compared the practice to an Eagles song — saying, "You can check out any time you like, but you can never leave." https://www.washingtonpost.com/news/powerpost/paloma/the-technology-202/2019/03/13/the-technology-202-freshman-sen-josh-hawley-emerges-as-one-of-toughest-republican-critics-of-big-tech/5c88136a1b326b2d177d6069/
Apr. 2	-	-
May 9	Rep. Jim Jordan (R-Ohio), Judiciary	During the Judiciary panel session, Republicans used their time to defend Barr's name and tried to divert the conversation to the origins of the Russia investigation, accusing the FBI of being guided by anti-Trump bias. "I think it's all about trying to destroy Bill Barr because Democrats are nervous that he's going to get to the bottom of everything," said Rep. Jim Jordan (R-Ohio). https://www.washingtonpost.com/politics/barr-to-trump-invoke-executive-privileged-over-redacted-mueller-materials/2019/05/07/51c52600-713e-11e9-b5ca-3d72a9fa8ff1_story.html
Jun. 5	Rep. Jerrold Nadler (D-NY), Judiciary	"The power of the state should not be used by one segment of society to impose its moral or religious beliefs on another," https://www.washingtonpost.com/arts-entertainment/2019/06/04/busy-philipps-testifies-about-abortion-rights-capitol-hill/
Jul. 24	Sen. Elizabeth Warren (D-MA), Armed Services	Sen. Elizabeth Warren (D-Mass.), a presidential hopeful, grilled Esper about his decision during his Senate Armed Services Committee confirmation hearing, arguing that if he would not commit to better distance himself from Raytheon, "you should not be confirmed as secretary of defense." https://www.washingtonpost.com/national-security/senate-votes-to-confirm-mark-esper-as-defense-secretary/2019/07/23/694b18a0-acb4-11e9-a0c9-6d2d7818f3da_story.html
Sep. 12	Rep. Jamie Raskin (D-MD), Oversight and Reform	"You can't tell me why there's a new policy. You can't tell me what motivated the new policy. And you can't tell me what the policy is," said Rep. Jamie Raskin (D-Md.), chairman of the House Oversight Committee's civil rights and civil liberties subcommittee. "Is that a correct assessment?" https://www.washingtonpost.com/immigration/administration-provides-few-answers-for-critically-ill-immigrants-facing-possible-deportation/2019/09/11/4e98d390-d4d1-11e9-9610-fb56c5522e1c_story.html
Oct. 24	Rep. Rashida Tlaib (D-MI), Financial Services	Rep. Rashida Tlaib (D-Mich.) raised those concerns during yesterday's hearing, saying Facebook permits a "lower standard for truthfulness and decency" for politicians, saying: "It is hate speech, it's hate, and it's leading to violence and death threats in my office." https://www.washingtonpost.com/news/powerpost/paloma/the-technology-202/2019/10/24/the-technology-202-mark-zuckerberg-struggles-to-defend-facebook-s-civil-rights-record/5db0811f602ff10cf14f9700/
Nov. 11	-	-
Dec. 5	Rep. Jerrold Nadler (D-NY), Judiciary	"I will honor my oath, and as I sit here today, having heard consistent, clear and compelling evidence that the president has abused his power, attempted to undermine the constitutional role of Congress, and corrupted our elections, I urge my colleagues to stand behind the oath you have taken," https://www.washingtonpost.com/politics/impeachment-hearings-live-updates/2019/12/04/b7cc7b4e-1682-11ea-a659-7d69641c6ff7_story.html

*The Washington Post based in D.C. ranks the top 5 in terms of circulation in the U.S. producing world-class news on politics (See https://www.agilitypr.com/resources/top-media-outlets/top-10-daily-american-newspapers/.) I chose 2019 because it is the most recent full calendar year that I can easily access manually on the Washington Post website, and I also tried to avoid the year 2020 in which the congressional session was abnormally affected by the outbreak of COVID-19. I chose ten days for the analysis by random sampling ten numbers from 1 to 31 with repetition using R: one in each month excluding January and August in which Congress is in recess. I had to adjust the order of assigning each number to each month to avoid choosing weekends in certain months.

Fig. S1. News Articles on Hearings and Members' Grandstanding Statements by Date

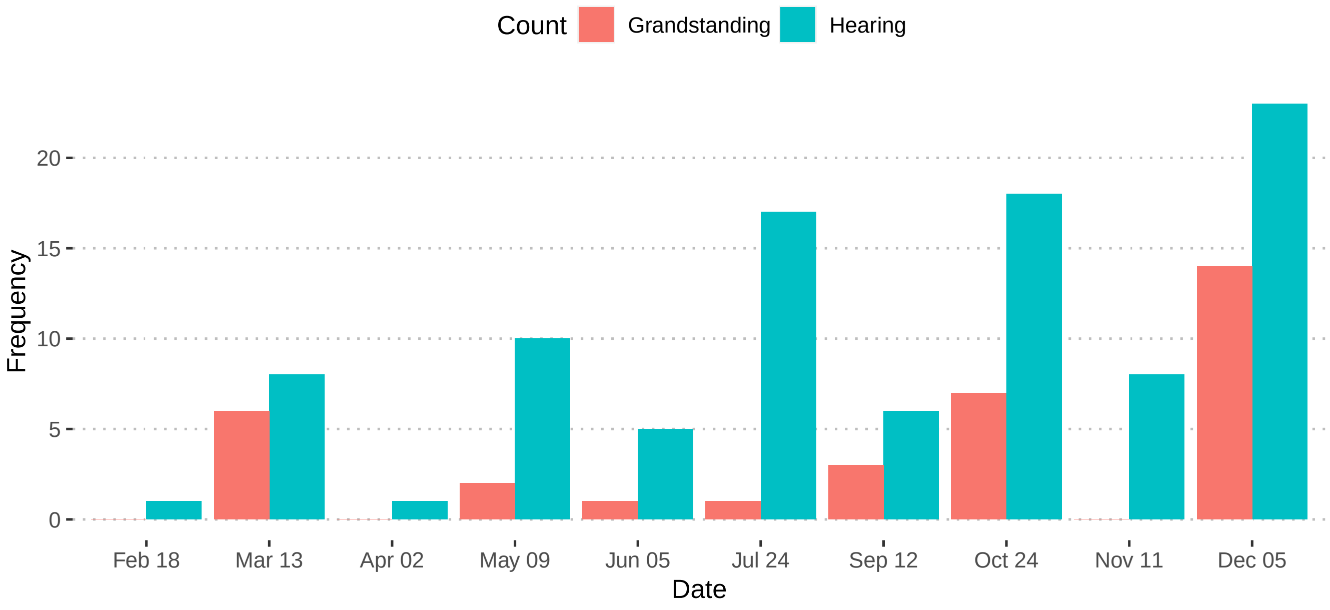

Second, beyond conventional communication channels, recently, politicians actively use social media platforms such as Twitter or YouTube to directly advertise their grandstanding moments in hearings.[†] Most of the members of Congress have their official Twitter handles and YouTube channels. Even congressional committees have them and post videos of hearing sessions in full or in part. One of the good examples that shows how politicians use these social media to send political messages to the public is shown in Figure S2. This is a tweet with a video clip in which Alexandria Ocasio-Cortez, a Democratic House Representative of New York, condemns Republicans for their anti-environmental move at a House Financial Services Committee hearing on environmental justice.[‡] This video clip was originally excerpted from the YouTube channel of the committee and tweeted by a social media user @nowthisnews. Then, the video clip quickly went viral as soon as she retweeted it on her own wall.[§] The view count of this video clip is 9.4 million, and it was retweeted more than 5,800 times and liked by more than 20,000 users.

In addition, conducted a more comprehensive analysis of congressmen's tweets. Among the 12,080 tweets that congressmen posted during the 115th Congress using their official Twitter accounts and contain the word "hearing", I randomly selected 100 tweets for the analysis. I find 88 of them are about congressional committee hearings and 48 of those about hearings contain either the video clip for a hearing session or members' statements made during the hearings they mentioned.

To better assess how many video clips containing congressional hearing sessions congressmen post for public view, I collected YouTube data. Using the official YouTube channels of the members of the 115th Congress provided by GovTrack.us, I extracted information of the video clips that a random sample of 96 members posted during the 115th Congress.[¶] Around 354 (8%) out of the 4,455 video clips they posted cover public hearing sessions typically including the member's speech, and these videos were viewed 1,516,094 times in total.[‖] Given that the selected channels constitute only one fifth of all members of the 115th Congress, approximately, I expect that 1,788 YouTube videos covering hearing sessions are published during this two-year period and the view count of all these videos would count over 7 million.

Besides newspapers and social media platforms, there are lots of diverse communication channels through which the statements that members make in hearings can be delivered to voters. To address this, I additionally collected 74,294 documents that contain public statements of members of the 114th Congress (2015-2016) from the Vote Smart Project website.[**] The original sources of these statements include press releases, statements that members post on their official websites, campaign websites or committee websites, newspaper articles, interviews, newsletters, press conferences, floor speeches, etc.[††] I find that 5,982 of the documents mention "hearing" in their main texts. Since the term "hearing" can be used differently other than referring to congressional hearings, I randomly sampled 100 documents that include "hearing" and identified false positives in which "hearing" is used to mean more generally "listen to someone." It turns out that 88 documents mention congressional hearings, and 33 documents directly quote or explain what members said in hearings. In addition, slightly less than half of them, 14 out of the 33, correspond to the definition of "grandstanding" used in this study. Given that, approximately 1,974 (33%) of the documents mentioning "hearing" are likely to convey statements that members made during congressional committee hearings, and approximately 837 (14%) grandstanding statements that members made in congressional hearings are published for voters' consumption in two years.[‡‡]

These analyses together suggest that what members said in committee hearings can reach their constituents through various public platforms as they are reproduced by the members themselves, committees, and the media.

[†] Only recently most of the members started using social media as their communication channel. So, the usage of this channel applies only to the last several congresses analyzed in this study. Indeed, when I add an interaction between the Grandstanding Score and the congress number treated as a numeric variable to Model 2 in Table 1 in the main text, I find a positive and statistically significant coefficient, 0.016 (s.e.= 0.008; p-value=0.039), suggesting that the effect of members' grandstanding on their vote share is stronger in more recent congresses. For example, the marginal effect of the Grandstanding Score in the 105th Congress is -0.001 but it increases to 0.145 in the 114th Congress. This is when the congress fixed effects are controlled as in Model 2, but dropping the congress fixed effects barely changes the result.

[‡] https://twitter.com/nowthisnews/status/1176563951712096256

[§] However, she deleted her posting from her account on December 13, 2019 according to the PolitWoops website: https://projects.propublica.org/politwoops/user/AOC?page=2

[¶] Originally, 100 members were randomly selected from the pool of both senators and House representatives, but four channels of them no longer existed.

[‖] These video postings are identified if either the title or the description of the video contains the word "hearing". Unlike other media data analyzed in this section, almost all video clips that mention "hearing" are about congressional committee hearings with few false positive cases.

[**] For further information about the data, please visit http://www.votesmart.org/

[††] I include all types of documents in the analysis except for congressional hearing transcripts and social media. There are only nine documents collected from social media in this data.

[‡‡] Note that although the documents from the Vote Smart Project include 2,595 newspaper articles, it does not seem to include all newspaper articles conveying members' statements published in the U.S. during the two-year period. In addition, I conducted more comprehensive search using Lexis-Nexis across various news outlets, but the search results frequently included contents about committees and hearings of European legislatures even with regional restrictions applied to the search criteria. Thus, I decided to choose a major U.S. news outlet and focus on it: the Washington Post.

Fig. S2. An Example Tweet with a Member's Grandstanding Moment Originally Excerpted from the House Finance Committee's YouTube Channel

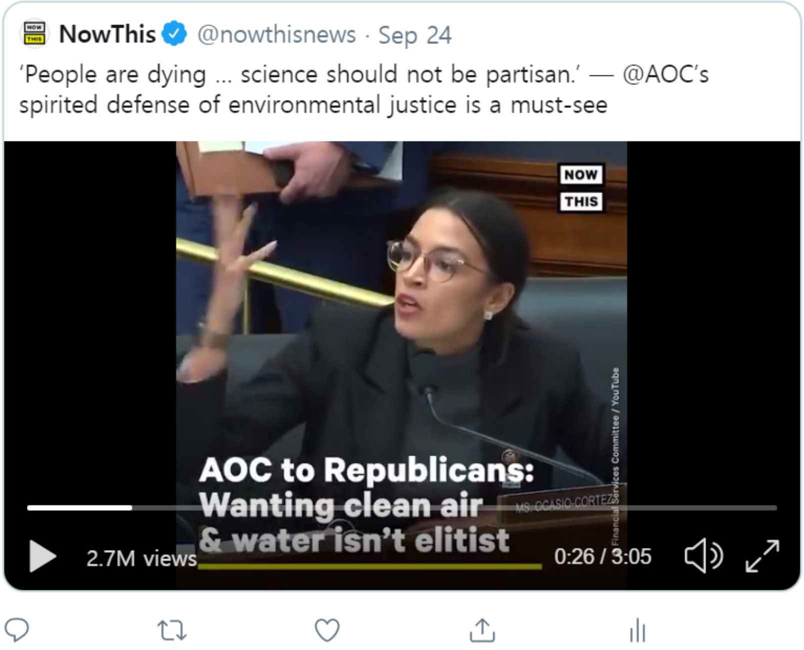

Fig. S3. Congressmen's Public Statements by Document Type

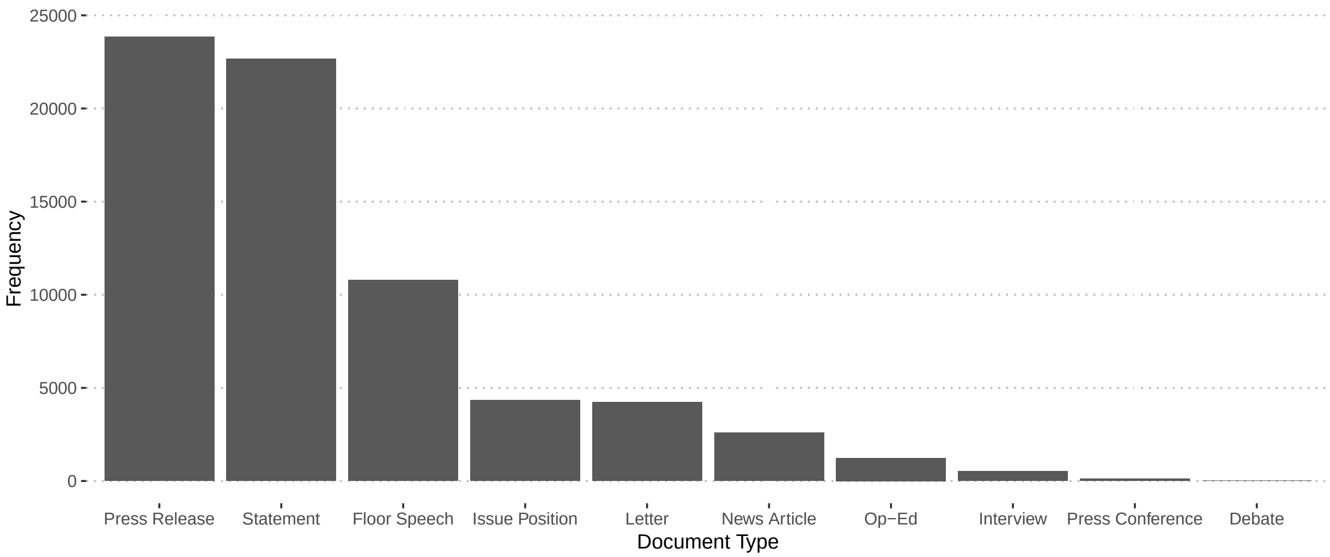

2. Validity of the Grandstanding Score

The Grandstanding Score is both statistically and substantively validated in Park (1). This section summarizes the validation strategy and results.

A. Statistical Validation. First, the statistical validation was conducted through the following procedure. From the 3000 sample paragraphs that were manually coded by online coders, only the 2700 randomly selected paragraphs are used to train machine learning models and the rest of the 300 are set aside as a validation set.

Park constructed three document matrices from the 2700 sample paragraphs in the training set. For each document matrix, she fit 13 machine learning models on the 2700 paragraphs including Random Forest, Lasso, Support Vector Machine, Kernlab's Support Vector Machine, Gradient Boosting Machine, Discrete Bayesian Additive Regression Tree sampler, Bayesian Generalized Linear Model and Linear regression with varying parameters for the first four models, which resulted in 39 model predictions for each paragraph in total. Then, she used an Ensemble Bayesian Model Averaging model which assigned non-zero weights to 9 of the 39 models and combined them. The following are the nine selected models:

1. Bag of words x Support Vector Machine
2. Bag of words x Kernlab's Support Vector Machine (epsilon = 0.1)
3. Bag of words x Kernlab's Support Vector Machine (epsilon = 0.5)
4. Bag of words x Gradient Boosting Machine (shrinkage = 0.01)
5. Tf-idf x Gradient Boosting Machine (shrinkage = 0.01)
6. Doc2vec x Kernlab's Support Vector Machine (epsilon = 0.1)
7. Doc2vec x Kernlab's Support Vector Machine (epsilon = 0.5)
8. Doc2vec x Lasso (nlambda = 100)
9. Doc2vec x Lasso (nlambda = 200)

Using the final ensemble model, she predicts the 300 paragraphs in the validation set. She compared the scores measured based on the online coders' manual coding with the scores predicted by the ensemble model to compute the root-mean-squared-error (RMSE). The RMSE is 0.613, and it is less than the RMSE of any of the single machine learning model out of the 39 models which ranges from 0.631 to 1.391. In addition, the Pearson's correlation coefficient between these scores is 0.703 while the same best performing single model produced the correlation of 0.673. This is consistent with the previous literature that claims that the ensemble method tends to have a stronger prediction power than any individual learning algorithm (Montgomery et al. 2012; Opitz and Maclin 1999; Rokach 2010).

In terms of the workers' quality, it was closely analyzed and monitored to achieve high quality work following the practice introduced in Carlson and Montgomery (2). Specifically, only 500 to 1000 comparisons were posted online at a time, which constitutes a batch. After retrieving each batch of work, poorly performing workers, defined as the ones providing answers deviant from other workers, were identified by running a random utility model which estimates both the SentimentIt score as well as the worker quality parameter for each worker. Then, the poor workers whose quality parameter estimates are below 1 are banned from further participating in the project. Also, note that in the process of computing the SentimentIt score, the workers' decisions are weighted by their quality. (See Carlson and Montgomery (2) for more details.)

The inter-coder reliability is less relevant in this project because the paragraphs to be compared in each pairwise comparison task are randomly matched. Thus, coders worked on different pairs rather than the same set making it challenging to compute the conventional inter-coder reliability. However, I could identify 298 duplicate pairs out of over 30,000 comparisons, and there were 149 unique pairs. Thus, each of the 149 were presented only twice in total. On 91 out 149 pairs (61%), the coders agreed.(2) conducted the same analysis using 50 movie reviews. In their project, workers were asked to choose a review which has more positive tone. They found 1049 duplicates with 423 unique comparisons done more than once in their data. The 73.8% of the duplicate cases had unanimous agreement among coders. Compared to their work, the agreement rate is lower for the Grandstanding Score probably because the concept that is measured is more complicated than simply assessing the positive tone in a text. Thus, more intense training of machine learning models was required, and all of these efforts are reflected in the validation statistics of the Grandstanding Score.

B. Substantive Validation. Second, the substantive validation takes several approaches. The first method is to compare the most frequent 200 words of the statements in the bottom quartile of the Grandstanding Score and those in the top quartile after removing the most frequent 50 words. The full list of these words is in S2. Those in italic are the words in both lists, but note that the frequency of these overlapping words in both types of statements can be different. The statements in the top quartile are featured by making "point"(-s) on "problem"(-s) and "concern"(-s) on the most salient, divisive issues such as "secur"-(ity), "tax", "health" "care" and "drug" frequently mentioning the "presid"(-ent), "administr"(-ation) and emphasizing adjectives and adverbs. On the other hand, the non-grandstanding statements tend to refer to the words relevant to information-seeking: "inform"(-ation), "expla(i)n"(-ation), "report"(-s), "record"(-s), "research", "data", "studi", "estim"(-ates), "assess"(-ment), "percent(-ages)" and "rate"(-s).

Table S2. Featured Words in Grandstanding vs. Non-grandstanding Statements

Type	Most Frequent Word Stems
Grandstanding	countri, problem, realli, congress, said, tri, concern, american, good, system, help, secur, lot, even, fact, put, first, job, money, administr, provid, bill, law, point, last, mean, busi, forward, may, understand, service, someth, hope, percent, believ, care, fund, cost, sure, health, part, unit, continu, act, depart, presid, public, happen, area, number, give, done, got, kind, interest, made, certain, appreci, inform, chang, wit, respons, million, support, report, legisl, agenc, whether, differ, hous, protect, two, seem, process, great, everi, plan, polici, abl, tell, increas, move, day, budget, find, community, actual, deal, compani, subcommitte, world, case, place, tax, littl, call, effort, particular, better, statement, pay, clear, billion, market, address, u., includ, start, develop, industri, regul, must, still, discuss, effect, opportun, agre, general, small, energi, creat, requir, person, offic, secretari, open, comment, anoth, reason, record, might, district, term, benefit, yield, allow, live, mayb, famili, long, home, without, drug, rule, propos, end, econom, sinc, order, level, keep, economi, colleagu, account, america, author, major, product, repres, meet, school, around, side, dollar, hold, answer, technolog, privat, best, spend, decis, exampl, resourc, current, panel, ago, forc, oper, futur, heard, impact, financ, real, local, yet, bring, manag, focus, pass, result, educ, insur, cut, togeth, consum, direct, critic, invest, addit, individu
Non-grandstanding	record, ms, dr, statement, wit, okay, correct, minut, panel, answer, next, may, first, offic, submit, open, inform, understand, 5, provid, report, cost, object, servic, pleas, requir, comment, part, depart, percent, appreci, without, ok, general, director, give, number, last, process, addit, fund, plan, agenc, gentleman, welcom, second, two, made, system, differ, respons, order, includ, mention, secretari, start, consent, specif, current, unanim, written, regard, secur, term, littl, help, yes, act, associ, manag, chair, presid, quick, point, million, recommend, believ, tell, u., subcommitte, might, area, said, request, concern, compani, rule, law, increas, administr, budget, project, follow, actual, review, data, begin, develop, bill, propos, abl, respond, oper, place, whether, forward, good, base, legisl, standard, impact, kind, move, within, case, chang, contract, busi, receiv, studi, author, day, guess, sir, direct, implement, discuss, relat, bit, address, effect, research, testify, technolog, final, mayb, polici, anoth, health, put, coupl, support, assist, rate, meet, billion, level, indic, turn, agre, mean, done, public, sinc, regul, proceed, chief, risk, explain, product, tri, rais, allow, call, determin, estim, interest, line, 10, 1, awar, three, 2, industri, posit, share, privat, earlier, unit, repres, sure, employe, someth, letter, vote, execut, refer, round, center, problem, benefit, heard, avail, improv, possibl, assess, list, rank, board, consid

The second method is to analyze the statements with the highest and lowest Grandstanding Scores. Below I present the top 5 and bottom 5 statements. (See the online appendix of Park (1) for more examples.) The most grandstanding statements tend to promote members' opinions on policies, criticize a head person of an executive body or grill a witness while the least grandstanding statements tend to delve into details on a policy issue and ask of witnesses' expectation on policy consequences, etc. Based on these analyses, the Grandstanding Score seems to successfully capture key characteristics of grandstanding statements and distinguishes them from non-grandstanding statements.

The Five Most Grandstanding Statements. [1] " And even the environmental movement should realize that the worst polluters in the world were the Socialist and Communist governments. People take better care of their own private property than is taken of property that is in public ownership. I haven't been to this area, but I bet these people take good care of their property. It is so sad to think that a lot of these people, or most of them, are people who came from Cuba where land was confiscated and taken away from them by a Communist dictator, and now they come to the United States, a country that is supposed to be a free country, yet these people are coming all the way from south Florida to Washington, DC, to try to defend their little piece of the American dream. The unbelievable thing is, this is happening all over this country. These liberal, left-wing, Socialist, big-government types think they can run everybody's life better than they can themselves. So they don't mind coming in and doing a really cruel thing and taking away this property. And I just think that we need to speak out against it and that it needs to stop, or at least hopefully we can slow it down a little bit. Thank you."

[2] " Let me just say that this was a half-a- billion-dollar mistake and I would ask that we point out that there was an action taken by your body under a justification of a legal definition that I think is a threat to both Democrats and Republicans that the word "is" is, and that for somebody to sit there and ignore the law and redefine the word "is" I think the American people are outraged that a half-a-billion-dollar issue was raised while legal jargon was ignoring the fact that the law is in there. And I don't think Democrat or Republican wants to have to add in every law that it will never happen. I yield back to the gentleman." [3] " And were never willing to admit just how oppressive they really were. I think it is time that we insisted that they face this reality of what Marxism/Leninism is all about. I think it is time that–there are many millions of Muslims around the world, many if not most who could be friends and be open to these kind of ideas of accepting people and not oppressing somebody simply because they worship God in a different way. We need to call to task the Saudis and the Pakistanis and these other people who have supposedly been on our side and quit trying to treat them with kid gloves because it ain't going to work. These regimes are basically gangster regimes in terms of the way they treat their people and it shouldn't be tolerated and the United States has done that. Shame on us. Thank God for you and Chris Smith and other people who have committed their lives to exposing those people who are stepping on the religious freedom of other human beings. Thank you very much for being with us today, and I think I am supposed to gavel this down. This hearing is now adjourned."

[4] " But I want to say one thing, though. I remember watching on television, as many people have. And it was really heart-rendering and warm when I saw the replica of the Statue of Liberty and everything it stood for. And all I can say is when we saw the tanks rolling in and as they brought the–basically, I understand they were drugged, hyped-up people from outside of Beijing in to roll over those people as they were screaming for help and screaming for the rest of the people to cry out for freedom, I mean, I just–I–it broke my heart. And for all those that are still fighting for freedom in China, they represent one-quarter of this world population. And yet we seem to turn our backs on them again and again. And I am glad that the people here in this room stand up for freedom and stand up for those, and I will always remember that photograph. Yield back the balance of my time. Thank you."

[5] " Right. And it is difficult, especially right now when we are having trouble recruiting, to walk away from people with a genuine love for their country. Obviously, it is not a policy. And to turn away from people who have done nothing wrong and to choose others who have committed some offenses and have been arrested for offenses and to say you are somehow better than others simply because of who people are–I am embarrassed. I mean, there is not a whole lot more to say except that I apologize that we use the wrong yardstick to measure a person's worth and devotion to the country. And it is my fervent hope that in 15 or 20 years we will change. Because I will tell you for myself that I may be straight, but I am not narrow. And I think that this policy here is very, very narrow. Thank you, and I yield back. I am sorry, may I take that one question, Chairwoman?"

The Five Least Grandstanding Statements. [1] " Dr. Boskin, I wanted to ask you, how would you go about assessing a risk adjusted rate of return? Mr. Cavanaugh in his written testimony talked about an adjusted risk rate of return. What would your comments be on that, and then I want to ask also Mr. Cavanaugh?"

[2] "Thank you. I would ask unanimous consent that the three statements previously submitted to the committee be entered into the record. Without objection, so ordered. Mr. Waxman, do you have any followup questions?"

[3] " Without objection, the witness's full statement will be inserted into the record. And, of course, each of you all are summarizing your statements. I want to thank Dr. Barth. At this time, our second witness is Ms. Janice Ayala, deputy assistant director of the Office of Investigations at United States Immigration and Customs Enforcement at the Department of Homeland Security. Welcome, Ms. Ayala."

[4] " Now, moving back to previous items of discussion, the SMRs. Just in general, how many applications do you anticipate receiving over the next couple of years, Dr. Lyons?"

[5] " OK. Thank you. And, Mr. Bardee–or, excuse me, Mr. Cauley, you noted that FERC has recently approved the NREC Critical Infrastructure Protection Version 5 standards which become enforceable on April 1 of next year, related to cybersecurity. First question is, can you briefly expand on the new Version 5 cybersecurity standards?"

Third, in addition to the validation of the score presented in Park (1), here I conduct one more analysis to demonstrate that the media tend to report members' grandstanding statements. For the analysis, I chose a hearing from the 114th Congress, which is the most recent congress in this data set, that was widely perceived as a hearing designed for political grandstanding: The Benghazi hearing held by the Select Committee investigating the issues around the Benghazi terrorist attacks on October 22, 2015 where then-Secretary of State Hilary Clinton was invited as a witness. Then, I collected news articles on this hearing published on the day of hearing and the day after from two major US news outlets: The Washington Post and Fox News. 14 articles were relevant to this hearing: 7 from each outlet. Then, I manually extracted members' statements that were quoted in these news articles and identified members' statements that include these quotes from the hearing transcript data set. I found 12 statements in total. Then, I conducted a two-sample t-test that compares the average Grandstanding Score of these statements (68.37) and the average Grandstanding Score of the rest of the statements that members made in this hearing (43.96). The gap between the two is statistically significant (p-value: 0.000019). This analysis suggests that members' statements scoring high in the Grandstanding Score tend to be perceived as newsworthy by journalists as well, which provides additional substantive validation of the Grandstanding Score.

3. Additional Regression Results

A. Main Results. This section provides full regression results that generated Table 1 and Figure 3 in the main text. In particular, Table S3 is a full version of Table 1 in the main text; Figure S4 presents two regression results based on which Figure 3 was drawn.

Table S3. The Effect of Grandstanding on Vote Share and PAC Contributions

	Vote Share (%)		*Vote Share (%, Lag)*	*PAC Contributions ($1K)*	
	Model 1	Model 2	Model 3	Model 4	Model 5
Grandstanding score	0.098**	0.070**	-0.001	0.367	0.692
	(0.043)	(0.031)	(0.042)	(1.166)	(1.392)
Statement frequency		4.997***	4.360		-290.301***
		(1.737)	(3.516)		(85.699)
Legislative effectiveness		-2.434	-0.754		591.541***
		(1.690)	(2.396)		(122.577)
Party support		8.662**	8.665*		47.438
		(4.236)	(4.890)		(124.729)
Seniority		5.389	28.535***		1,013.378***
		(7.914)	(9.134)		(393.237)
Seniority squared		-0.234***	-0.603***		-2.052
		(0.064)	(0.091)		(2.878)
Ideological intensity		6.615**	-6.586*		-157.059
		(2.659)	(3.667)		(118.544)
Committee leader		0.586*	-0.012		22.609
		(0.328)	(0.564)		(14.879)
Party leader		0.130	1.585*		54.015
		(0.595)	(0.947)		(34.489)
Minority		6.309**	1.566		199.436
		(2.894)	(3.340)		(138.449)
Unified		1.381	9.830*		352.174
		(4.456)	(5.244)		(219.621)
Minority*Unified		-11.102*	-12.914*		-405.260
		(5.881)	(6.936)		(282.649)
Redistricted		-0.652	2.390**		32.025
		(0.649)	(1.142)		(21.001)
Secure district		17.730***	39.979***		-49.821
		(5.694)	(5.461)		(152.155)
Challenger quality		-2.362***	-0.648		-10.122
		(0.329)	(0.418)		(13.716)
Spending ratio		13.257***	4.524***		23.791
		(1.163)	(0.988)		(26.375)
President party		-6.872**	-9.139***		-144.833
		(2.902)	(3.362)		(141.018)
Midterm		4.535*	-4.292		-212.463*
		(2.426)	(2.766)		(120.374)
President party*Midterm		-2.756***	1.560**		-60.461***
		(0.478)	(0.660)		(17.625)
Log of primary candidates					-20.792***
					(7.757)
Constant	56.884***	36.031***	49.692***	382.819***	424.763**
	(2.188)	(5.328)	(6.218)	(44.140)	(197.052)
Member effect	Fixed	Fixed	Fixed	Fixed	Fixed
Congress effect	Fixed	Fixed	Fixed	Fixed	Fixed
Observations	3,259	2,401	2,471	3,183	2,474
Adjusted R^2	0.504	0.748	0.651	0.607	0.631

Note: *p<0.1; **p<0.05; ***p<0.01

Robust standard errors clustered by member are in parentheses.

Table S4. Moderating Effects of Salience and Redistricting (Statement-level)

	Vote Share (%)	
Grandstanding score	-0.002***	0.002***
	(0.0004)	(0.0003)
Salience	-0.870***	-0.190
	(0.205)	(0.190)
Statement frequency	6.872***	6.875***
	(0.069)	(0.069)
Vote share	-0.017***	-0.017***
	(0.006)	(0.006)
Vote share squared	0.0002***	0.0002***
	(0.00004)	(0.00004)
Legislative effectiveness	-3.295***	-3.311***
	(0.077)	(0.077)
Party support	11.484***	11.467***
	(0.196)	(0.196)
Seniority	6.282***	6.266***
	(0.371)	(0.371)
Seniority squared	-0.337***	-0.337***
	(0.003)	(0.003)
Ideological intensity	3.610***	3.606***
	(0.142)	(0.142)
Committee leader statement	-0.020*	-0.018
	(0.012)	(0.012)
Party leader	-0.180***	-0.182***
	(0.042)	(0.042)
Minority	-3.411	-3.415
	(5.001)	(5.002)
Unified	-5.724	-5.723
	(5.006)	(5.006)
Minority x Unified	7.590	7.594
	(10.003)	(10.004)
Redistricted	-0.399***	-0.343***
	(0.041)	(0.048)
Secure district	11.044***	11.038***
	(0.205)	(0.205)
Challenger quality	-1.973***	-1.973***
	(0.016)	(0.016)
Spending ratio	14.408***	14.406***
	(0.038)	(0.038)
President party	2.483	2.486
	(5.001)	(5.002)
Midterm	2.627***	2.626***
	(0.195)	(0.195)
President party x Midterm	-3.872***	-3.873***
	(0.026)	(0.026)
Grandstanding x Salience	0.015***	
	(0.002)	
Grandstanding x Redistricted		-0.001**
		(0.0006)
Constant	40.098***	39.956***
	(0.380)	(0.380)
Observations	664,649	664,649
Note:	*p<0.1; **p<0.05; ***p<0.01	

Congress and committee fixed effects and hearing and legislator random effects are included. Standard errors are in parentheses.

B. Additional Analysis Controlling for Unit-specific Linear Trend. To additionally control for time-varying member-specific confounders that might not have been captured by the variables included in the main analysis, this section replicates the analysis controlling for the unit-specific linear time trend. For this, I take two approaches: using fixed effects and random effects. First, interactions between the member fixed effects and the time variable are added to the list of controls in the full model. To capture time, the congress's number is used as a numeric variable. In this model, 579 more parameters are estimated. Second, an alternative way to control for the unit-specific time trend without sacrificing the degrees of freedom is to allow a random slope for the time variable to vary by member.[§§]

Table S5 present results for these two regression models. In the first model addressing the unit-specific time trend using fixed effects, the coefficient on the Grandstanding Score is not statistically significant. However, in the second model, the Grandstanding Score has a positive and statistically significant coefficient. Thus, these tests provide mixed results.

Table S5. The Effect of Grandstanding on Vote Share with the Unit-Specific Linear Trend

	Dependent variable:	
	OLS	*Mixed-effects*
Grandstanding score	0.047	0.063**
	(0.035)	(0.032)
Statement frequency	3.607*	4.839**
	(1.885)	(2.250)
Legislative effectiveness	-2.027	-2.350
	(1.818)	(2.028)
Party support	3.994	9.146**
	(4.481)	(3.818)
Seniority	62.950***	8.210
	(15.508)	(10.103)
Seniority squared	-2.167***	-0.295***
	(0.665)	(0.085)
Ideological intensity	8.136***	8.027**
	(3.082)	(3.169)
Committee leader	0.755*	0.688
	(0.388)	(0.422)
Party leader	0.113	0.043
	(0.804)	(0.798)
Minority	-26.754***	-0.349
	(8.487)	(0.480)
Unified	-30.527***	
	(9.341)	
Minority*Unified	55.140***	2.427***
	(17.057)	(0.768)
Redistricted	-0.260	-0.388
	(0.721)	(0.762)
Secure district	6.941	10.313**
	(4.610)	(4.008)
Challenger quality	-1.731***	-2.261***
	(0.333)	(0.338)
Spending ratio	12.454***	12.808***
	(1.198)	(0.792)
President party	26.817***	
	(8.549)	
Midterm	-0.977	
	(0.910)	
President party*Midterm	-3.325***	-2.861***
	(0.485)	(0.530)
Constant	541.433***	40.165
	(117.652)	(96.513)
Member effect	Fixed	Fixed
Congress effect	Fixed	Fixed
Member-specific Trend	Fixed	Random slope
Observations	2,401	2,401
Adjusted R^2	0.795	
Note:	*p<0.1; **p<0.05; ***p<0.01	

In the first model, robust standard errors clustered by member are in parentheses. In the second model, standard errors are in parentheses. Coefficients for the following variables were not estimated in the second model: Unified, President party and Midterm.

Why does the result in the first model addressing all the member-specific variations using fixed effects differ from the results of other model

[§§] In this model, only the random slope is introduced without a random intercept for each member because member fixed effects are already included in the model. However, I also tried adding a random intercept for each member, but the coefficients and standard errors for fixed effect variables including the Grandstanding Score do not change.

specifications? There can be several potential reasons for this.

First, almost 80% of the members in my data served only one, two, three, or four terms during the period that the data covers. Thus, controlling for a unit-specific linear time trend might have soaked up too much variation in the vote share of these short-lived members leaving little room to be explained by other substantively important covariates. Table S6 below presents the distribution of the number of terms that members served in the House during the period covered in this data.[¶¶]

Table S6. Number of Terms Each Member Served from the 105th to 114th Congresses

Number of Terms	1	2	3	4	5	6	7	8	9	10	Total
Number of Members	224	186	120	107	74	41	25	18	8	1	804

In Model 2 presented in the main text, those who served only a single term are dropped as they do not allow analysis of within-member variations over time. When the unit-specific linear trend is controlled, among the 580 members used to fit Model 2, the 186 members who served only two terms are perfectly explained by the linear trend. Thus, only the 394 members who were observed three or more times contribute to estimating the effects of other covariates, and 57.6% of them served for only three to four terms. Compared to other studies where the same cases are observed for 10-20 times with almost no attrition problem across the cases (3), this data contain little variation to be explained by other variables after the unit-specific linear trend is controlled using a fixed effects approach. Thus, this approach can be less suitable for this highly imbalanced short panel data.

Second, compared to Model 2 in the main text, in this model, several substantively important variables lost statistical significance: the Grandstanding Score; party loyalty, which captures how often a member votes along their party line on party unity votes; and secure district, which measures the level of partisan homogeneity of a member's district based on their votes in the most recent presidential election. It is important to note that, theoretically, these three variables are likely to move together in a similar direction as well as with the member's vote share such that members with constituents more homogenously leaning toward one party tend to enjoy higher vote shares, grandstand more often, and vote along the party line. Thus, insignificant effects of these three variables do not necessarily mean that these variables have no effect on or no relationship with members' vote share. Rather, the unit-specific linear time trend effectively absorbed the effects of these variables that are moving in a similar direction. Unfortunately, this adds further difficulty to analyzing which of these variables are affecting another by covering their relationship under one large blanket. One such relationship can be the effect of members' grandstanding on their vote share.

Third, the degrees of freedom are largely reduced by controlling for the unit-specific linear trend using the fixed effects approach which requires estimating 579 more parameters. Currently, 1,408 parameters are estimated based on 2,401 observations used to fit the model. Extant literature suggests that around ten times more observations are needed to fit one additional parameter (4, 5). Given that, the degrees of freedom of the model are extremely limited.[***] The lack of degrees of freedom renders the distribution of t-values to have thicker tails, and thus the p-values for coefficients on covariates may grow larger in general. Then, the model is more likely to induce false negatives.

In summary, the set of analyses presented in this section generated mixed results. However, given the statistical and theoretical reasonings described above and the analyses reported in the main text showing that reverse causality is unlikely, the overall findings imply that members' grandstanding efforts have a correlational effect on their vote share.

4. Mechanism Analysis

A. Theory and Analysis. This section provides an additional analysis exploring through which micro-level mechanism members' grandstanding increases their vote shares. Theoretically, I propose that there are two principal ways to increase an incumbent's chances of reelection. The first is to increase the turnout of in-partisans. If constituents who voted in the previous election do not change their vote choices, the only way to increase the incumbent's vote share is to mobilize supporters who did not vote previously to turn out to the extent that its increase is greater than any increase in the turnout rate of non-supporters.[†††] However, the turnout of both groups may decrease in midterm elections and increase in presidential elections. Technically, then, as long as in-partisans are relatively better mobilized than independents or out-partisans compared to the previous election after accounting for the dip in the midterm election, the incumbent's vote share may increase. Indeed, partisan messages are known to increase voter turnout especially among base supporters (6). Therefore, political messages that members send out are likely to better mobilize in-partisan voters and increase the incumbent's vote share in the following election.

The second way that an incumbent's reelection chances may increase is through persuading independents or opposite partisans to switch their votes in favor of the incumbent. Theoretically speaking, if turnout rates of both supporters and non-supporters are fixed, the only way that an incumbent's vote share can increase is to win votes from those who did not support the incumbent in the previous election. Politicians' position-taking messages are found to have a persuasion effect by shifting public opinion (7). Studies note that voters tend to be persuaded by a like-minded candidate's messages (8) but dissuaded by an unlike-minded candidate's messages, and the size of the dissuasion effect is larger than that of the persuasion effect (9). However, the persuasion effect among strong in-partisans and the dissuasion effect among strong out-partisans are unlikely to affect their vote choices and will not contribute to vote shifts. Instead, vote shifts can be induced by a persuasion effect on independents or lean out-partisans and a backfire effect on lean in-partisans. As long as the former trumps the latter, the incumbent's grandstanding is likely to have a positive persuasion effect and increase their vote share in the following election.

However, note that these two mechanisms–mobilization and persuasion effects– may work simultaneously. This section tests whether any of these mechanisms are at play and, if so, to what extent. I test the following two hypotheses:

Mobilization Hypothesis: As a member grandstands more in any given congress, in-partisan supporters in their district are more likely than independents or out-partisans to be mobilized to turn out in the following election.

Persuasion Hypothesis: As a member grandstands more in any given congress, independents or out-partisans are likely to switch their votes in support of them.

For the analysis, I use the Cooperative Election Study (CES)'s three-wave panel survey data from 2010 to 2014 which include surveys fielded biannually covering three elections held during this period. Note that the same survey respondents were interviewed for three times in this panel survey data.

For the analysis, I selected cases according to the following criteria. First, as in the main analysis, only the cases in which an incumbent ran for reelection are considered. Second, one major challenge in using this short panel data is that there was a large-scale redistricting in January 2012. Because the effect of grandstanding changes upon redistricting, as shown in the main analysis, probably due to sudden disconnection between incumbents and constituents, only the respondents in the districts unaffected by the 2012 redistricting are included.[‡‡‡] Third, when estimating within-individual effects, the cases where the match between an incumbent and a respondent is observed only in one wave out of the three are automatically dropped from a regression model. Thus, I drop these cases from the data.

[¶¶] The member-level hearing data include 949 unique members. However, excluding observations that have missing values in any of the variables used in the full model specifications leaves 804 members.

[***] Model 2 in the main text did not have enough degrees of freedom either, but estimating 70% more parameters can certainly make a difference to the estimation of the model.

[†††] Out-partisans can be mobilized by the incumbent's partisan messages: a backfire effect.

[‡‡‡] Even though the match between an incumbent and a respondent did not change, politicians whose district boundary changed might have modified their policy-positions or ways of communication to accommodate new constituents. In addition to these changes, other unidentified district-level environments influencing the relationship between an incumbent and a respondent might have changed upon redistricting. Thus, including the incumbent-respondent pairs in the redistricted regions in this analysis will introduce time-varying, unobservable confounders. Therefore, the analysis excludes responses collected from the districts that experienced redistricting in the 2012 survey.

The data include multiple variables that allow testing various mechanisms. Theoretically, of my particular interest are respondents' validated turnout[§§§] and whether they said they voted for the incumbent. Given the errors and biases in self-reported vote choices documented in previous literature (11) and especially low response rate for the vote choice question, however, to complement the analysis, I also test the effect of members' grandstanding on respondents' partisan affiliation with the incumbent party. All these three dependent variables are binary indicators.[¶¶¶] The unit of analysis in this section is an individual respondent, and I look at the over-time changes in each of the respondents.

The main explanatory variable is the incumbent's Grandstanding Score, and I control for the following potential confounders. At the respondent-level, I control for age, the level of education, family income, the level of political interest and binary indicators for registered voters and unemployment.[17] At the member-level, I include the Legislative Effectiveness Score (LES), seniority and its squared value, challenger quality, campaign spending,[18] previous vote share, and binary indicators for party leaders and minority members.[19] Then, in the model, respondent fixed effects and congress fixed effects are included. As the dependent variables are binary, I use a fixed-effects logit model (a.k.a. conditional logistic regression) using "clogit" function in "survival" R package (13).

Table S7 presents regression results. The first model tests the mobilization hypothesis which proposes that there can be a mobilization effect especially among in-partisans rather than among independents and out-partisans. The Grandstanding Score is interacted with partisan groups with independents as a reference group. The result shows that there is neither a mobilization effect nor a demobilization effect in any group.

The second model tests the persuasion hypothesis, that is, whether incumbents' grandstanding tends to motivate non-supporters more likely to vote for the incumbent. However, it finds no evidence of vote switches in any group. Even without the interaction with partisan groups, the politicians' grandstanding tendency does not have any independent effects on voter turnout or vote choices. (See Table S8 for the results.)

These results are inconsistent with the member-level findings presented in the main text. The potential reason for this inconsistency might be related to a typical issue of survey respondents' self-reported vote choices. By using the validated turnout, the first model overcomes any potential bias in self-reported behavior. Yet, the second model on candidate choices still suffers from this problem. Their vote choices can never be validated for the nature of the secret ballot system. So, the result in the first model on turnout is relatively more reliable than that in the second model on vote choices. This suggests that there is no mobilization effect but there is still a possibility of vote switches resulting from members' grandstanding and the vote switches may not have been fully captured in the respondents' self-reported vote choices. Therefore, the partial mismatch between the aggregate-level election results and micro-level survey results presented in this analysis suggests that the revealed behavior observed through aggregate-level electoral results is likely be a better measure of voter preferences than expressed behavior observed through surveys. This analysis, thus, further generates an important methodological implication for studying mass behavior.

The third model in Table S7 examines the effect of grandstanding on voters' affiliation with the incumbent party. Interestingly, I find that as the incumbent grandstands more, the respondents are more likely to switch their partisanship to affiliate with the incumbent party. The coefficient on the Grandstanding Score is positive and highly significant. Given that voters who switched partisanship to the incumbent's party are also likely to switch their votes to the incumbent, this result provides indirect support for the persuasion hypothesis.

Then, why do we not find respondents' vote switches while we observe their partisanship switches as well as the increased vote share of the incumbents resulting from members' grandstanding? I identify two potential reasons. First, survey respondents often misreport their vote choices.(11) Due to the secrecy of vote choices, misreporting can be worse for the vote choice question than for any other questions including the turnout. Second, not only misreporting vote choices, but also high non-response rates can be another reason. Despite the anonymity of the survey data, respondents may be reluctant to reveal their vote choices compared to any other survey items. Indeed, the non-response rate for this item is 13.1% as opposed to 3.6% for the turnout question and 1.8% for the question on partisanship.[20] Thus, the self-reported vote choices may suffer from selection bias in terms of who responds and who do not. In this panel regression analysis, only the observations that changed over time contribute to the fit of the regression. So, if those who switched their votes feel uncomfortable revealing their vote choices and refuse to respond at a higher rate than those who did not switch their votes, the regression result which relies only on the vote switchers will fail to correctly estimate the relationship between members' grandstanding and voters' vote choices. Below I provide a simple test on this argument.

To test whether there was such a selection, I investigate if the respondents who shifted their partisanship and thus might have changed their vote choices are less likely to reveal who they voted for while they still report their partisanship. Indeed, I find that the non-response rate for the vote choice question among those who switched their partisanship from an out-partisan to an in-partisan or vice versa between elections (15.29%) is higher than the non-response rate among those who did not (13.36%) although the difference between the two groups is not statistically significant based on the two-sided z-test of two-sample proportions. However, also note that the difference between the two groups is over 4% (13.96% vs. 9.83%) and statistically significant at 0.015 level when all responses in including the ones in redistricted districts in 2012 are used. This result substantiates my claim that the null effect of the members' grandstanding behavior on the respondents' vote choices may be related to the tendency that those who changed their vote choices are more likely to shy away from reporting their vote choices. In addition, the respondents who did not switch their partisanship yet but only switched their votes in favor of the incumbent are less likely to report their vote choices than those who switched both the partisanship and vote choices at the same time with more confidence. However, the former cannot be identified. Thus, this test on the latter group serves as a weaker test of the former group.

To be completely free from any potential intervention of the 2012 redistricting, a cleaner test can be conducted using only the responses from the single-district states such as Alaska. In this dataset, there are 308 such cases from five states. The five states are Alaska, Delaware, South Dakota, Vermont and Wyoming. The same regression model specifications are used including only these responses. The regression results are presented in Table S9. The results show that out-partisans tend to switch their votes to the incumbent as the incumbent grandstands more. The coefficient on the interaction term between the Grandstanding Score and the indicator for out-partisans is positive and highly significant, and the marginal effect of the Grandstanding Score for this group is positive. The analysis further shows no effect of the incumbent's grandstanding on turnout and partisanship. Although the results are slightly different from those shown in Table S7, they corroborate the evidence for the persuasion effect.

Then, why would the incumbents' grandstanding induce vote switches among their constituents who did not support the incumbent in the previous election? There can be multiple ways how message contents make some voters switch their votes in favor of the incumbent. For example, the incumbent's policy stance can be adopted by message receivers (7); the incumbent's presentation of self may increase their valence or likability that voters perceive (14, 15); the same may be at work at the party-level rather than at the individual member-level, from the perspective of message politics (16), such that voters may evaluate the incumbent party more highly or favorably as the messages contain partisan messages. However, testing each of these psychological mechanisms is beyond the scope of this analysis.

In summary, the findings presented in this section suggests that the increase in the vote share of the grandstanding incumbents can be explained by voters' vote switches to the incumbent party rather than the mobilization effect. This is consistent with Spenkuch and Toniatti's finding that TV campaign advertisements have a persuasion effect but do not affect voters' turnout.(17)

[§§§] I only use validated voters because self-reported turnout is often found untruthful in previous studies (e.g., (10)), and I confirmed that the current dataset suffers from this issue as well.

[¶¶¶] Except for the turnout record, the other three dependent variables were originally ordered variables, but I converted them to binary variables for the following reason. There are neither many ready-made statistical packages that estimates a fixed-effects ordered logit model consistently nor a consensus on the validity of a few new approaches to estimate the estimators. One option recently made available is the "feologit" command in Stata (12), but none of my model specifications were fit due to non-convergence.

[17] The coding rules are presented in the next section.

[18] First of all, I control for the effect of campaign spending in three different forms depending on the dependent variable. As in the main analysis where the dependent variable was members' vote share, I control for the spending ratio between the incumbent and challenger when the dependent variables are survey respondents' vote choices and partisanship choices because these choices are made in zero-sum competition. However, voters' turnout is likely to increase as the absolute amount of campaign spending from all candidate's increases. Thus, in the turnout model, I control for the spending of both candidates and spending of the incumbent, respectively.

[19] The interaction between the indicators for president party members and midterm elections is not included. First, because the president did not change during the period covered in this analysis, due to the absence of variations within respondents, the coefficient is not estimated for this variable. Second, the midterm indicator is same as the fixed effect for the 112th Congress.

[20] However, it makes sense that less people would refuse to respond to the vote choice question because only those who turned out to vote can say who they voted for. Still, 4.4% out of those who said they voted did not report their vote choices.

Table S7. The Effects of Grandstanding on Voter Behavior (2009-2012)

	Turnout	Vote Choice	Partisanship
Grandstanding score	−0.0001	0.0049	0.0074**
	(0.0002)	(0.0082)	(0.0031)
In-partisan	−0.0167	0.4787	
	(0.0149)	(0.3869)	
Out-partisan	−0.0162	−0.4992	
	(0.0126)	(0.8467)	
Age	0.0022	−0.0097	0.0389
	(0.0027)	(0.0387)	(0.0448)
Education	0.0001	−0.1972**	−0.0168
	(0.0004)	(0.0979)	(0.0179)
Registered voter	−0.0034	−0.3896	−0.0120
	(0.0044)	(0.3213)	(0.0451)
Political interest	−0.0041	−0.0235	−0.0177
	(0.0051)	(0.0381)	(0.0470)
Unemployment	−0.0084	0.0146	−0.0361
	(0.0062)	(0.0344)	(0.0410)
Family income	−0.0003	−0.0002	0.0021
	(0.0003)	(0.0049)	(0.0039)
Legislative effectiveness	0.0058	−0.0193	0.3373*
	(0.0122)	(0.1224)	(0.1871)
Seniority	−0.0535	0.5474	0.0516
	(0.0342)	(0.3464)	(0.5393)
Seniority squared	−0.00004	−0.0096*	0.0009
	(0.0005)	(0.0057)	(0.0073)
Party leader	0.0109**	0.0092	−0.0483
	(0.0054)	(0.0480)	(0.0349)
Minority	0.0017	0.0063	−0.0231
	(0.0017)	(0.0189)	(0.0246)
Vote share$_{(t-1)}$	−0.0010*	−0.0011	0.0018
	(0.0006)	(0.0028)	(0.0028)
Challenger quality	−0.0015	−0.0282	−0.0479*
	(0.0029)	(0.0268)	(0.0288)
Spending both	−0.0000		
	(0.0000)		
Spending ratio		0.0192	0.0492
		(0.0737)	(0.0569)
112th Congress	0.0675*	−0.0073	0.0023
	(0.0393)	(0.0293)	(0.0298)
113th Congress	0.0003	0.0117	−0.0132
	(0.0010)	(0.0232)	(0.0339)
Grandstanding	0.0004	−0.0038	
x In-partisan	(0.0003)	(0.0086)	
Grandstanding	0.0004	0.0005	
x Out-partisan	(0.0003)	(0.0192)	
Respondent effect	Fixed	Fixed	Fixed
Observations	8,221	7,911	8,857
Log Likelihood	−2,837.9160	−1,987.1590	−3,178.2430

*p<0.1; **p<0.05; ***p<0.01

Note: All dependent variables are binary indicators. Robust standard errors are reported in parentheses.

Table S8. Additional Analysis on the Effects of Grandstanding on Voter Behavior

	Turnout	Vote for Inc.
Grandstanding Score	0.0002	0.0020
	(0.0001)	(0.0023)
In-partisan	−0.0006	0.3089**
	(0.0011)	(0.1217)
Out-partisan	−0.0002	−0.4769*
	(0.0013)	(0.2871)
Age	0.0030	−0.0116
	(0.0029)	(0.0406)
Education	−0.0001	−0.1978**
	(0.0004)	(0.0978)
Registered voter	0.0029	−0.3947
	(0.0034)	(0.3267)
Political interest	−0.0040	−0.0252
	(0.0051)	(0.0364)
Unemployment	−0.0099	0.0145
	(0.0063)	(0.0340)
Family income	−0.0002	−0.0005
	(0.0003)	(0.0046))
Legislative effectiveness	0.0105	−0.0084
	(0.0136)	(0.1204)
Seniority	−0.0456	0.5458
	(0.0289)	(0.3464)
Seniority squared	0.0007	−0.0095*
	(0.0008)	(0.0058)
Party leader	0.0098*	0.0058
	(0.0051)	(0.0472)
Minority	0.0005	0.0069
	(0.0011)	(0.0194)
Vote share$_{(t-1)}$		−0.0013
		(0.0028)
Challenger quality	−0.0012	−0.0293
	(0.0028)	(0.0291)
Spending both	0.0000	
	(0.0000)	
Spending ratio		0.0207
		(0.0740)
112th Congress	0.0640*	−0.0065
	(0.0378)	(0.0292)
113th Congress	−0.0021*	0.0121
	(0.0012)	(0.0231)
Respondent effect	Fixed	Fixed
Observations	8,221	7,911
Log Likelihood	−2,837.9220	−1,987.1690

Note: Robust standard errors are reported in parentheses.
*p<0.1; **p<0.05; ***p<0.01

Table S9. The Effects of Grandstanding on Voter Behavior (Single-district States Only)

	Turnout	Vote for Inc.	In-Partisanship
Grandstanding Score	0.1528	0.0644	0.0025
	(0.4021)	(0.1757)	(0.0840)
In-partisan	2.3086	−3.7886	
	(1.4686)	(9.3182)	
Out-partisan	0.4967	−77.3734***	
	(0.9809)	(18.1476)	
Age	0.0959*	0.0909	0.0077
	(0.0512)	(0.0838)	(0.0134)
Education	0.0834	−0.0199	−0.0316
	(0.0581)	(0.0618)	(0.0224)
Political interest	−0.0740	0.0333	0.0181
	(0.1172)	(0.0351)	(0.0142)
Unemployment	−0.8465	−0.0119	−0.3061
	(0.5161)	(0.0570)	(0.1970)
Family income	0.0059	−0.0082	0.0012
	(0.0169)	(0.0098)	(0.0042)
Legislative effectiveness	−7.3188	−6.8864	0.1545
	(20.2494)	(8.6363)	(4.1358)
Seniority	0.2523	−1.0875*	0.1213
	(2.2175)	(0.6097)	(0.4346)
Seniority squared	0.1150	0.1170	0.0025
	(0.3579)	(0.1495)	(0.0728)
Party leader	−0.1503	−0.0839	0.0347
	(0.6403)	(0.1879)	(0.1271)
Minority	−0.7746	−0.8071	0.0065
	(2.1042)	(0.9430)	(0.4326)
Grandstanding x In-partisan	−0.0650	0.0838	
	(0.0404)	(0.2058)	
Grandstanding x Out-partisan	−0.0170	2.0838***	
	(0.0258)	(0.4883)	
Respondent effect	Fixed	Fixed	Fixed
Observations	208	219	244
Log Likelihood	−142.3399	−127.2657	−129.8340
Note:		*p<0.1; **p<0.05; ***p<0.01	

Note: Robust standard errors are reported in parentheses. The coefficients for the following variables were not estimated and thus not reported in the table: Registered voter, vote share$_{(t-1)}$, challenger quality, campaign spending variables, and indicators for the 112th and 113th Congresses.

That being said, however, the exploration of the mechanisms presented in this section is not the final word to how members' grandstanding works in voters' mind but was intended to provide the first analysis using the best possible available observational data. Given the nature of the data and the research design used in this analysis, the findings presented here are necessarily correlational. Future research involving a randomized experiment may be able to further confirm the causal path and extend the investigation of the micro-level mechanisms.

B. A Customized Codebook for the CES Panel Survey Data. Validated turnout: 1 for validated voters based on the Catalist data; 0 for validated non-voters.
Vote choice: 1 if the voter self-reported to vote for the incumbent in the post-election survey; 0 for voting for a challenger.
Partisanship: 1 for affiliation with the incumbent party; 0 otherwise.
In-partisan: 1 for affiliation with the incumbent party; 0 otherwise.
Out-partisan: 1 for affiliation with the opposition party; 0 otherwise.
Age: 1 for below 30; 2 for 30-39; 3 for 40-49; 4 for 50-59; 5 for 60 or older.
Education: 1 for no high school education; 2 for high school graduate; 3 for some college education; 4 for two years of college education; 5 for four years of college education; and 6 for postgraduate education.
Registered voter: 1 for a registered voter; 0 otherwise.
Political interest (Following what's going on in government and public affairs): 1 for hardly at all; 2 for only now and then; 3 for some of the time; 4 for most of the time.
Unemployment: 1 for unemployed; 0 otherwise.
Income: Family income in an 18-point scale with 1 for less than $10,000 and 18 for $25,000 or more.

References

1. JY Park, When do politicians grandstand? measuring message politics in committee hearings. *J. Polit.* (2021).
2. D Carlson, JM Montgomery, A pairwise comparison framework for fast, flexible, and reliable human coding of political texts. *Am. Polit. Sci. Rev.* **111**, 835–843 (2020).
3. A Fouirnaies, H Mutlu-Eren, English bacon: Copartisan bias in intergovernmental grant allocation in england. *The J. Polit.* pp. 805–817 (2015).
4. F Harrell, *Regression Modeling Strategies.* (Springer International Publishing), (2016).
5. RD Riley, et al., Minimum sample size for developing a multivariable prediction model: Part ii - binary and time-to-event outcomes. *Stat. Medicine* **38**, 1276–1296 (year?).
6. F Foos, EA de Rooij, The role of partisan cues in voter mobilization campaigns: evidence from a randomized field experiment. *Elect. Stud.* **45**, 63–74 (2017).
7. DE Broockman, DM Butler, The causal effects of elite position-taking on voter attitudes: Field experiments with elite communication. *Am. J. Polit. Sci.* **61(1)**, 208–221 (2017).
8. JN Druckman, On the limits of framing effects: Who can frame? *J. Polit.* **63(4)**, 1041–1066 (2001).
9. M Gilens, N Murakawa, Elite cues and political decision making. *Polit. Decis. Making, Deliberation Particip.* **6**, 15–49 (2002).
10. MW Traugott, JP Katosh, Response validity in surveys of voting behavior. *Public Opin. Q.* **43**, 359–377 (1979).
11. J Wright, Contributions, Lobbying, and Committee Voting in the U.S. House of Representatives. *Am. Polit. Sci. Rev.* **84**, 417–438 (1990).
12. G Baetschmann, A Ballantyne, KE Staub, R Winkelmann, feologit: A new command for fitting fixed-effects ordered logit models. *The Stata J.* **2**, 253–275 (2020).
13. TM Therneau, *A Package for Survival Analysis in R*, (2020) R package version 3.2-7.
14. RF Fenno, *Home Style: House Members in Their Districts.* (Longman), (1978).
15. GA Huber, K Arceneaux, Identifying the persuasive effects of presidential advertising. *Am. J. Polit. Sci.* **51**, 957–977 (2007).
16. FE Lee, *Insecure Majorities: Congress and The Perpetual Campaign.* (The University of Chicago Press. IL.), (2016).
17. JL Spenkuch, D Toniatti, Political advertising and election results. *Q. J. Econ.* pp. 1981–2036 (2018).
